# Legal issues in the implementation of Maternal Death Surveillance and Response: a scoping review

**DOI:** 10.1093/heapol/czae071

**Published:** 2024-08-03

**Authors:** Mulu Beyene Kidanemariam, Ingrid Miljeteig, Karen Marie Moland, Andrea Melberg

**Affiliations:** Faculty of Law, University of Bergen, Magnus Lagabøtes Plass 1, Bergen 5010, Norway; School of Law, Mekelle University, Adi-Haqi Campus, Mekelle, Tigray, Ethiopia; Bergen Center for Ethics and Priority Setting in Health (BCEPS), University of Bergen, Aarstadveien 21, Bergen 5009, Norway; Centre for International Health, University of Bergen, Aarstadveien 21, Bergen 5009, Norway; Bergen Center for Ethics and Priority Setting in Health (BCEPS), University of Bergen, Aarstadveien 21, Bergen 5009, Norway; Centre for International Health, University of Bergen, Aarstadveien 21, Bergen 5009, Norway

**Keywords:** MDSR, maternal death, death review, legal issues, privacy, health workers, scoping review

## Abstract

The Maternal Death Surveillance and Response (MDSR) system is designed to continuously identify and review all maternal deaths. It aims to help countries understand the scale and distribution of maternal deaths, identify their causes, and inform corrective measures to address the challenge. Despite the growing adoption of the MDSR by numerous low- or middle-income countries, its implementation faces various challenges, including legal ones. This scoping review was conducted to map legal issues and challenges that arise during the implementation of the MDSR. It adapted the Bain and Kongnyuy framework, categorizing legal issues into data, people, use of findings, and legal regulation. Literature was retrieved from seven databases, complemented by additional online searches. We included studies published in English between 2010 and November 2022 that report on legal issues arising during the implementation of MDSR. Out of 1174 studies screened, 31 were selected for review. The review highlighted the limited attention given to the legal dimension of the MDSR by the research community. It also documented the lack of adequate legal framework essential for the system’s effective implementation. Inadequate safeguards for informational privacy and the lack of confidentiality reinforce a prevalent sense of being blamed, mainly among health workers. Consequently, widespread under-reporting and intentional misattribution of causes of maternal death, defensive referrals, and disengagement from the MDSR process were reported. We recommend that implementing countries regulate the gathering and use of MDSR data through appropriate laws and legally ensure that the MDSR data are only used for the intended purpose. Appropriate complaint-handling mechanisms are needed in health systems to prevent the misuse of the MDSR. Future studies on MDSR implementation would benefit from involving legal experts, considering the multifaceted legal dimensions of the MDSR.

Key messages

- There is a scarcity of empirical studies documenting the legal issues in Maternal Death Surveillance and Response (MDSR) implementation.
- Prevalent legal issues that the MDSR faces relate to informational privacy, confidentiality, and fear of blame and litigation among health workers. Legal regulation for MDSR should aim to address these issues.
- The lack of legal frameworks supporting MDSR implementation is apparent.
- The ‘data, people, use of findings, and legal regulation’ framework could be utilized in future research to explore specific laws impacting the MDSR.

## The MDSR: an introduction

Maternal mortality remains a significant global public health challenge. According to a recent report, ‘in 2020 an estimated 287000 women globally died from maternal causes, equivalent to almost 800 maternal deaths every day…’ (WHO, 2023). Historically, maternal mortality became a global issue in the mid-1980s when studies revealed that the maternal mortality rate in many African countries was much higher than previously thought (WHO, 1986; De Brouwere *et al*., 1998; Starrs, 2006). International initiatives for the recognition of women’s rights and reproductive health rights in the 1990s, coupled with the growing evidence that most maternal deaths are preventable, led to further recognition of maternal mortality as a human rights issue. Today, maternal mortality is used as an indicator not only of the strength of health systems and gender equity but also of compliance with certain human rights (De Brouwere *et al*., 1998; Cabal and Stoffregen, 2009; Aasen, 2013; Yamin, 2013).

Understanding the scale, trend and distribution of maternal mortality and identifying causes and contributing factors to maternal deaths have been fundamental in efforts to address the challenge (WHO, 2015). However, many countries burdened by high maternal mortality lack functional systems to generate reliable data. This poses an obstacle to the development and monitoring of effective policies and interventions to mitigate the problem (Gaimard, 2014). This deficiency became apparent during the periodic progress assessment of the Millennium Development Goals in 2010. It was determined that many countries lacked information on maternal health, contributing to the slow progress towards meeting the Millennium Goals to reduce maternal and child mortality. The lack of reliable data also affected global funding and accountability mechanisms (Yamin and Falb, 2012).

In response, the World Health Organization (WHO) and other partners developed the Maternal Death Surveillance and Response (MDSR) system in 2013. The MDSR constitutes a continuous cycle of activities designed to identify, report and review all maternal deaths; establish causes and assess the preventability of death; and based on the data gathered, respond to failures identified to prevent similar occurrences in the future (Abebe *et al*., 2017; Smith *et al*., 2017a). It is thus often divided into four stages: identification and notification, review, analysis and recommendation, and response and monitoring. The primary aim of the MDSR is to guide policies and evaluate the effectiveness of interventions. As emphasized by the WHO, the goal is ‘not just estimating the number of maternal deaths to know the magnitude of the problem but also understanding why and where the women died to be able to do something about it’ (WHO, 2023). The MDSR also has the potential to serve as a tool for advocacy and accountability by drawing attention to preventable maternal death and providing data that can be used in assessing countries’ efforts towards their commitments to address preventable maternal death (WHO, 2013). It is noteworthy that the MDSR, later expanded to include perinatal deaths, is also referred to as Maternal and Perinatal Death Surveillance and Response (MPDSR) in the literature (WHO, 2016b; Lusambili *et al*., 2019).

Given its investigation-like features and the involvement of various actors in the process, the implementation of the MDSR often gives rise to certain legal concerns. During the death review process, for instance, open discussion among reviewers is required to establish the causes and preventability of the death in question. This heightens concerns about responsibility, particularly among health workers, as the process can potentially reveal incriminating information (Bain and Kongnyuy, 2018). The fact that health data are at the centre of the process also implies that the MDSR engenders important privacy rights. Moreover, the MDSR involves diverse stakeholders whose interests in the process do not always align (Østebø *et al*., 2018; Melberg *et al*., 2019; Strong, 2020). Among others, family and community members, health workers, facility managers, governmental organs, professional organizations, civil society, the media and relevant international organs participate in the MDSR in various capacities.

Anticipating such challenges, the MDSR Technical Guidance—developed by the WHO and its partners in 2013 to steer its implementation—advises adopting countries to establish appropriate legal frameworks (WHO, 2013). This is deemed crucial for the effectiveness of the system. More specifically, the guidance emphasizes conducting the MDSR in compliance with a ‘no name, no shame’ approach, highlighting the importance of safeguarding those participating in the MDSR process. It also recommends that countries establish legal mechanisms to prevent the use of the review findings in litigation (WHO, 2013). Moreover, it advises countries to safeguard the data collected for this purpose and to maintain the confidentiality of the process. Despite these stipulations, inadequate legal protection and prevalent naming and blaming remain among the challenges hindering MDSR implementation (WHO, 2014; 2016a; Smith *et al*., 2017b; Melberg *et al*., 2019; Kinney *et al*., 2021).

However, there is a dearth of research on how the legal challenges are manifested in practice (WHO, 2016a; Smith *et al*., 2017a; Bain and Kongnyuy, 2018). The WHO’s survey on the implementation of the MDSR in 67 countries concluded that it ‘found little literature that considers MDSR from a legal perspective’ (WHO, 2016a: p. 31). A comprehensive recent review on MDSR implementation in low- and middle-income countries, similarly, concluded that although the absence of a strong MDSR legal framework is contributing to fear among health workers, ‘explicit aspects of fear about litigation are not described or explored’ in the literature (Kinney *et al*., 2021: p. 966). This research aims to map the available literature on the nature and forms of legal topics and issues that arise in the implementation of the MDSR. The research question for this review is: What is known from the existing literature about legal issues that arise during the implementation of the MDSR?

## Conceptual framework

As a central component of the research question, the expression ‘legal issues’ requires clarification. Conventionally, a legal issue is discussed in the context of court cases, where it refers to the part of a dispute that necessitates the application of a specific law for its resolution. However, this understanding is too narrow for our purposes. A broader definition provided by Black’s Law Dictionary better reflects our interpretation in this review. Accordingly, a legal issue is defined as ‘of or relating to law; falling within the providence of law; established, required, or permitted by law…’ (Black and Garner, 2004, p. 912). ‘Issue’ in the context of the research question, in turn, often signifies a topic, subject matter, dispute or concern. An issue thus can be a mere topic involving, relating to, or regulated by law or a problem necessitating a legal solution (Neslund *et al*., 2010; Gostin and Wiley, 2016). Thus, in this review, legal issues include subjects regulated by existing law, involving legal institutions, or typically subject to legal regulation.

In the context of the MDSR, a legal issue may encompass MDSR activities governed by existing laws, those anticipated (e.g. in the Technical Guidance) to be regulated by law, matters related to legally protected interests or rights, situations likely to or empirically leading to issues traditionally addressed by legal regulations or cases requiring resolution by judicial or semi-judicial bodies within the relevant jurisdiction. Even if domestic statutory laws constitute the predominant form of laws governing public health (Parmet, 2009), ‘law’ in the context of legal issues in MDSR may also include relevant judicial decisions. Such a broad interpretation of the expression ‘legal issues’ aligns with the general nature of our research question.

To conceptualize potential legal issues relevant to the MDSR, we reviewed pertinent frameworks, including the MDSR cycle (WHO, 2016a), the Conceptual Implementation Framework for MPDSR (Kinney *et al*., 2019) and a list of topics discussed as ‘legal issues’ in selected public health surveillance literature (Neslund *et al*., 2010; Gostin and Wiley, 2016). A framework developed by Bain and Kongnyuy (2018) proved to be more suitable for our review due to its apparent relevance to discussions in the relevant literature and its relative effectiveness in minimizing overlaps across the categories proposed, as described later.

Bain and Kongnyuy (2018) classified legal issues that arise in the implementation of the MDSR into three categories: data, people and the use of findings. While potential legal issues about ‘data’ relate to rules and legal standards regulating the collection, use, storage or transfer of data used for MDSR, under ‘people’, the focus shifts to the extent to which protection is afforded to individuals and facilities participating in the MDSR from legal or administrative measures associated with their involvement in the process. Complementing this, the ‘use of findings’ pertains to whether and how the use of files and documents used for or developed during the MDSR process is confined only to the MDSR purposes, namely, understanding why women die so that preventive strategies can be developed.

With increased familiarity with the literature during the screening and charting stages, we noticed that some MDSR-relevant legal issues are not covered under the three categories. These include questions about the forms of tools governing MDSR systems and how the authority to carry out the various MDSR activities is granted. We thus added a fourth category, expanding the existing framework to better capture more legal dimensions of the MDSR. We propose to term this category ‘legal regulation’. The framework so developed is presented in **Box 1**.

Box 1: **Legal issues in MDSR: a conceptual framework**

**Data**

- Access to (personal) data (including collection, use and transfer);
- Ensuring the privacy/confidentiality of data collected;
- Guaranteeing anonymity and standardizing anonymization of data.

**People**

- Protection of individuals and institutions involved in the MDSR against legal, administrative or professional measures arising from or connected to their involvement in the MDSR process;
- Protection of review committee members from subpoena as witnesses in relation to their role in the MDSR;
- Instances of litigation or administrative proceedings associated with MDSR activities;
- Fear of litigation/punitive action associated with participation in the MDSR.

**Use of findings**

- Limiting the use of MDSR results or files gathered or produced in the MDSR process only for MDSR objectives;
- Separating the MDSR from legal or administrative punitive actions.

**Legal regulation**

- Laws/quasi-legal tools governing the MDSR or parts thereof;
- Laws mandating the establishment of the MDSR system, review committee(s) or other organs involved;
- Mandatory registration/notification of maternal deaths;
- Involvement of lawyers/legal experts in the MDSR process.

## Methods

### Developing a protocol

To guide the scoping review, we developed a protocol, following the Arksey and O’Malley approach, as further refined by the Joanna Briggs Institute Manual and others (Arksey and O’Malley, 2005; Levac *et al*., 2010; Aromataris, 2020; Peters *et al*., 2022). The protocol, which is annexed as Supplementary File 1, describes the method and justifies our choice of research design. It outlines how we intend to carry out the six stages of the review recommended by the Arksey and O’Malley approach, which are (1) identifying the research question, (2) identifying relevant studies, (3) study selection, (4) data collection, (5) data summary and synthesis of results and (6) optional consultations.

### Eligibility, information sources and search

We included all literature published or accessible online in English between 2010 and November 2022 that reports on legal issues arising during the implementation of maternal or MPDSR systems The year 2010 marks the starting point of policy initiatives that ultimately established the MDSR (Danel *et al*., 2011; WHO, 2016a). We limited the selection to literature discussing MDSR implementation to gauge the empirical experience of countries.

We excluded publications that exclusively deal with perinatal or neonatal review systems, as well as those solely discussing confidential enquiry into maternal death. Confidential enquiries were excluded due to their fundamental differences from the MDSR. Among others, unlike in MDSRs, reviewers in confidential enquiries are typically external bodies not affiliated with the facilities where deaths occur. Besides, such enquiries focus on facility deaths, and review is often conducted on selected cases. Furthermore, they are adopted in settings with functioning statistical infrastructure (WHO, 2004; Hussein *et al*., 2009; Moodley *et al*., 2014).

Assisted by university librarians, we initially tested our search strategy on PubMed and JSTOR and determined the search terms. While Table 1 summarizes the search keys, the full search history is provided in Supplementary File 2. In December 2022, we conducted a comprehensive search on Medline, Embase, Web of Science, PubMed Central, JSTOR, HeinOnline, and Westlaw UK databases. In addition, we conducted an open search using Oria, the search engine of the University of Bergen (UiB), Norway, to identify further studies, including grey literature. We selected the first 100 hits, based on the assumption that subsequent screening would yield few additional relevant works (Stevinson and Lawlor, 2004). Furthermore, we conducted a hand search on the WHO online library. Through June 2023, we also identified more publications from an alert system linked to Oria, which we had set up to track new publications. Finally, we reviewed the reference lists of selected papers for any further records.

**Table 1.** Overview of search key components

Summary of search terms	(“Maternal Death Surveillance and Response” or MDSR or “Maternal and Perinatal Death Surveillance and Response” or MPDSR). ((maternal death or perinatal death or neonatal death) adj2^2^ (surveillance or response or review or reporting or audit)).
Concept component	All forms of maternal and perinatal surveillance, review and response systems, regardless of nomenclature. Systems that contain elements of identifying, reporting, reviewing maternal death (or along with perinatal death) and responding to the deaths. Limited to studies that examine the implementation of the system at national and subnational levels or across countries. Excludes studies exclusively dealing with perinatal surveillance and response systems and confidential enquiries into maternal deaths.^3^
Context component	Limited to studies published in English between 2010 and November 2022.

### Study selection (screening)

To test the feasibility and ensure consistency in approach, M.B.K., A.M. and I.M. initially screened 15 titles independently. Subsequently, M.B.K. screened titles and/or abstracts of the records, and A.M. verified the results. Similarly, five records were randomly selected, and the three reviewers independently conducted a full-text review against the set criteria. During this process, we observed that several papers contained minimal data material to address our research question, and we decided to further limit our selection to papers that discuss at least one substantive legal issue, as described earlier. Following discussions and consensus, M.B.K. performed a full-text review of the records and A.M. verified the results. Discrepancies were resolved through discussion, involving I.M. A list of the studies selected is provided in the record index, Supplementary File 3.

### Data charting process

The research team (M.B.K., A.M., I.M. and K.M.M.) developed a data charting form based on the Bain and Kongnyuy framework, categorizing the legal issues around the MDSR into three sets: data, people and the use of findings. After pilot testing with five records and gaining increased familiarity with the literature, we found that the three categories leave out some aspects and issues of a legal nature relevant to the functioning of the MDSR. We, therefore, added the fourth category, ‘legal regulation,’ as described earlier.

Finally, M.B.K. charted the data and A.M. verified the results. All authors then discussed the findings. The charting form was continuously revised to reflect the review team’s consensus regarding the form and the results included. Data items charted included key reference characteristics and background, including authors’ names, year of publication, country of focus, the scale and level of study, research design and key relevant findings. The substantive findings were recorded into the four categories by relevance. A description of key characteristics for each included study is provided in Supplementary File 4, and key results are summarized in Table 2. For further details of results, see Supplementary File 5.

**Table 2.** Summary of results

Categories	Subcategories	Example of records	Summary of results
Data	Accessing data for MDSR	2, 3, 23	Through data sharing agreements, mandated by law
		5, 9, 21, 22	Problems of accessing health data from facilities or information systems
		5, 8, 26, 27	No formal mechanism of data sharing or mandate to access health information systems or vital event records
	Identifiable health data	4, 7, 28, 29	Names of the deceased, health workers and facilities
	Tools for data collection, transfer, and storing	5, 8, 10, 21, 22	WhatsApp, SMS, telephone, case notes, paper-based, software, database
	Time of anonymization	2, 22, 26, 27	After local review or after the report is published
	Lack or breach of anonymity/confidentiality	2, 4, 7, 8, 12, 16, 28	Anonymity and confidentiality are violated
		7, 21	The identity of health workers and facilities revealed
	Effect of breach	4	Poor dialogue during case reviews
	Solution suggested	12	Adopting laws
People Involved	Extent of the blame challenge	1, 2, 3, 6, 8,11, 15, 17, 18, 19, 24, 25, 27, 28, 29, 30	Reported widespread blame or fear thereof
	Factors reinforcing blame or fear thereof	15, 16, 23	Increasing malpractice cases
		1	Focus on institutional delivery inducing fear of reporting by community members
		1, 13	Strong political will to reduce maternal death
		3, 4, 11, 20	Lack of understanding about the process
		13, 16	Hostile media coverage
	Forms of blame experienced or feared	11, 15, 17, 29	Legal action, criminal and civil litigation, malpractice cases
		10, 17	Suspension from job
		17, 24	Referral to labour office, disciplinary committee
		10, 21, 24, 25	Oral and written warnings, reprimands
		24	Disciplinary action
		11	Feeling threatened
		2, 10, 13, 16, 19, 20, 29	‘Other punishments’, ‘punitive action’, negative consequences or repercussions
		2, 13	Having to explain to officials
		15	Inviting external investigation
		2, 4	Being called out during review, considered unskilled
	Targets of blame	1, 2, 6, 10, 11, 14, 15, 16, 20, 27, 28, 29	Health workers
		7, 15, 16, 19, 22, 25	Lower-level health workers, front-line workers, junior doctors
		20, 29	Traditional birth attendants
		15, 22, 28	Family members, community members, deceased women
	Effects of blame or fear thereof	1, 13, 15, 18, 19, 20, 25, 28, 29, 31	Failure to report, under-reporting, selective reporting
		19, 20, 25, 29, 31	Severe under-reporting of abortion-related maternal deaths
		2, 20, 21, 29	Avoiding reviews, resistance to speak freely
		6	Low review completion rate
		2, 15, 16, 20, 25, 28	Unnecessary referral, refusal to admit/treat patients
		2, 15, 16, 20, 28, 30	Misclassification, falsification of documents, misattributing cause of death
		2, 14, 25	Weak dissemination
		16, 28	Demotivating students from choosing obstetrics as a career
	Ways out adopted/ suggested to mitigate blame and its effects	1, 12, 24, 25, 27, 28, 30	Introducing legal protection, appropriate laws
		8, 18, 21, 25, 26, 27, 29, 30, 31	Training, orientation, sensitization, better information, commitment, assurance
		16	Indemnity insurance
Use of findings	Reported use of MDSR results for other purposes	21	For legal proceedings
		7, 15, 20	Reported connection between MDSR and disciplinary measures
		2	For administrative purposes
		1, 7,16, 18, 20, 22, 26, 28, 31	Understanding that it can be or is used in legal or disciplinary settings
	Insulating the MDSR from punitive measures by law	27, 28	Strictly separated by law
		14, 24	Call for insulation by law
Legal regulation	Mandatory notification of maternal death	1, 3, 5, 6,11, 14, 15, 16, 17, 22, 23, 26	Maternal death is a notifiable event, including as a public health emergency
		22, 23, 29	Mandated by law
	Regulatory deficit	1, 7, 8	Unclear mandates and obligations in MDSR
		2, 3, 4, 20, 31	Ambiguity about the purpose of MDSR
	Forms/tools of governing the MDSR or its parts	3, 5, 6, 7, 13	Ministerial order, decree, circular
		7, 10, 24, 26, 28	Audit charters, guidelines, codes of conduct, Memorandum of Understanding
	Need for legal framework	9, 10, 12, 31	Law/legal framework as an enabler
		2, 5, 11, 12, 22, 23, 26, 27, 28, 30	Absence of law as a weakness or impediment; calls for legal framework
		5, 13, 22, 30	Rules exist but are not complied with

### Data analysis

We analysed the data thematically using a deductive-inductive approach (Clarke and Braun, 2017). Accordingly, the data were initially organized into the four categories described earlier. Then, we inductively and iteratively developed subcategories from the gathered data sets, which formed the basis for reporting the results. We also provided a descriptive narration and numerical summary of the study characteristics as part of the process.

### Consultation

The protocol was presented on several occasions to gather feedback, including to members of a multi-country, multidisciplinary research project working on the MDSR system at the University of Bergen,^1^ in November 2022. It was also discussed in a legal methodology course in March 2023 and at an international seminar in May 2023, which was attended by experts in medical and public health, as well as health ethics. Preliminary results were shared at a MATRISET seminar in early November 2023. Additionally, expert librarians from both the faculties of Medicine and Law at the UiB were consulted for advice on search terms and strategies.

The review decision process is reported using an adapted ‘Preferred Reporting Items for Systematic Reviews and Meta-Analyses extension for Scoping Reviews’ Checklist (Tricco *et al*., 2018), available in Supplementary File 6.

## Results

### Included studies

**Figure 1.**
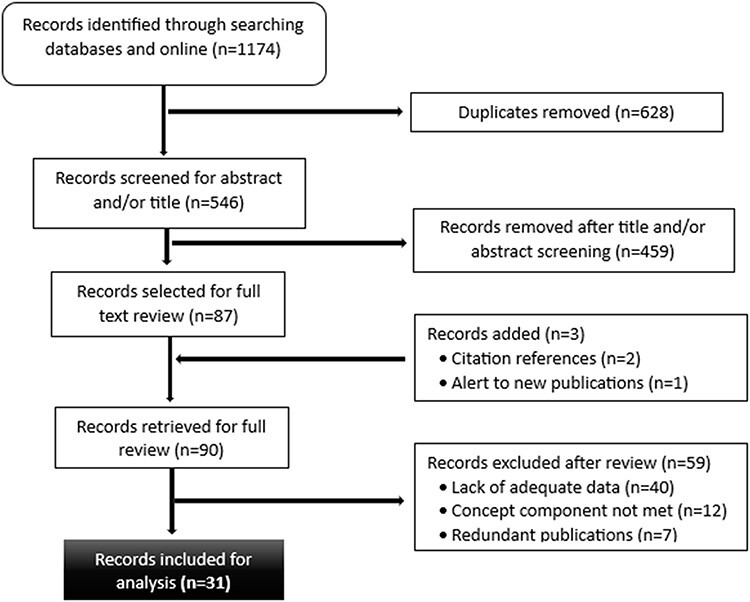
Flow diagram of the study selection process

The initial search resulted in a total of 1174 records. All citations were imported into the EndNote20 citation manager, where duplications were removed. After removing duplicates, we screened 546 unique results by title and/or abstract, yielding 87 publications eligible for a full review. An additional three records were identified—two from citation references and one from an alert to new publications. This led to a total of 90 records undergoing full-text review, of which 31 met the inclusion and exclusion criteria. Twenty-eight of the records were academic journal articles, and three were study reports. All the academic articles were published in journals dedicated to medical, public health or health sciences (20 in total). Figure 1 shows the flow diagram documenting the screening process, and Table 3 summarizes the number of search results by source.

**Table 3.** Result of search by source

Database/search engine	Number of records
PubMed Central	314
Embase	283
Web of Science	239
Medline (Ovid)	214
Oria, search engine for the University of Bergen	100
JSTOR	21
WHO website	3
Total records	1174

Of the 31 included records, 22 examined the implementation of the MDSR system in specific countries, covering a total of 17 countries. The rest reported the status of MDSR implementation in certain regions, a selection of countries or countries across the globe. Most individual countries represented were in Africa (13), two were in Asia, and the remaining two were in the Americas. In terms of the publication year, 18 records were published since 2019, and 11 were published between 2014 and 2018, with only two records published between 2010 and 2013. An approximately equal number of records examined the implementation of MDSR at both national and subnational levels. Most of the records (23) were qualitative studies, with the remainder employing mixed methods. While most of the records (27) discussed the full cycle of the MDSR system, a few of them (four) focused on one or more of the stages. Notably, none of the records exclusively or significantly discussed legal issues. Next, we present a summary of the results by category.

### MDSR data–related legal issues

This category encompasses legal issues and standards that relate to how data are collected, stored, used or transferred during the implementation of the MDSR. Numerous studies discussed various aspects of the data used in the MDSR and the challenges faced in accessing different information sources and systems. The literature focused on the safety of the MDSR data after its collection and is often discussed under ‘anonymity’ and/or ‘confidentiality’, without providing further definitional information. Even if the studies showed that the privacy rights of individuals are threatened in the process, particular privacy laws were rarely mentioned.

#### The nature and collection of MDSR data

Some studies described the nature of the data collected for the MDSR system, indicating that it includes personally identifiable information such as names and addresses of the deceased, health workers or institutions (Armstrong *et al*., 2014; WHO, 2014). Similarly, several studies described a variety of tools used in the collection, storage and sharing of such data. Accordingly, data are collected and shared among the actors involved through telephone calls, SMS, WhatsApp group chats, physical case notes as well as digitized databases (Kinney *et al*., 2020; Bongajum *et al*., 2021; Compaore *et al*., 2022).

In certain countries, such as Ethiopia and Chad, MDSR data were gathered through channels designed specifically for this purpose (Abebe *et al*., 2017; Abouchadi *et al*., 2022; Kouanda *et al*., 2022). In other instances, like in the state of Ohio in the USA, such data were acquired through linkage to existing health information systems (Shellhaas and Conrey, 2018). In the latter cases, access was facilitated through mutual understanding and goodwill among the relevant organs (Smith *et al*., 2017b) or by signing formal data sharing agreements, mandated by law (Agaro *et al*., 2016; Abouchadi *et al*., 2022). The availability of such laws that empower MDSR organs to acquire data from other sources supported the process. In contrast, some studies reported that the lack of formal data sharing laws or other systems negatively affected MDSR implementation, by making access to relevant data difficult (Goodman *et al*., 2013; Scott and Dairo, 2015; Bongajum *et al*., 2021; Said *et al*., 2021).

### Data privacy challenges

While none of the studies included addressed pre-data collection legal considerations, a few studies briefly described the forms and timing of anonymization or the mandated destruction of collected data. While some countries required data anonymization after a facility review is conducted, in others, the data could remain identifiable until a report is published (Armstrong *et al*., 2014; Melberg *et al*., 2020; Kodan *et al*., 2021). However, none of the studies discussed legal standards of anonymity.

Several studies report weaknesses in securing the privacy of the data gathered (Armstrong *et al*., 2014; Melberg *et al*., 2020; Kodan *et al*., 2021; Abouchadi *et al*., 2022). In a few instances, it was also indicated that the identity of health workers and facilities was deliberately disclosed to the public as a form of punishment for poor attendance and engagement in review sessions (Said *et al*., 2021). A study from Indonesia further noted that the MDSR was treated as a forum for performance evaluation, leading to the public disclosure of findings, including the identity of the health workers and the institution involved (Cahyanti *et al*., 2021). Such breaches of anonymity and confidentiality led to poor dialogue during death reviews (Kodan *et al*., 2021). Recommendations for the adoption of appropriate laws to ensure confidentiality and anonymity of the process were provided although they often lack specificity (WHO, 2016a; Kodan *et al*., 2021; Willcox *et al*., 2023).

### Fear of blame and inadequate legal protection

This category explores the forms, exacerbating factors and effects of concerns of a legal nature that actors involved in the implementation of the MDSR manifest in relation to their participation in the MDSR process. The studies highlighted a pronounced sense of vulnerability among participants, with a predominant theme of experiencing or fearing blame for maternal deaths. Health workers, who are the backbone of the MDSR process, emerged as the principal target of these concerns.

### The extent and forms of blame

Most studies reported a widespread association of the MDSR system with blame (Agaro *et al*., 2016; WHO, 2016a; Abebe *et al*., 2017; Smith *et al*., 2017a; Melberg *et al*., 2019; Naik *et al*., 2020; Kinney *et al*., 2021; Willcox *et al*., 2023). Broadly stated, it was believed that the way the MDSR was conducted and its outcomes were widely used, or were amenable, to placing blame on individuals and institutions involved in the process. Some studies mentioned practical experiences that justified the perception or fear of the respondents; however, the reasons were not clarified in many others.

The experience of blame took various forms. For example, the MDSR resulted in or was associated with court cases, including instances of criminal and civil litigations (WHO, 2014; Melberg *et al*., 2019; Mukinda *et al*., 2021), cases of work suspension (Kinney *et al*., 2020; Mukinda *et al*., 2021) and referral to human resource offices for a likely disciplinary action (Shittu and Kinney, 2017; Mukinda *et al*., 2021). Other forms of blame included oral or written warnings (Kinney *et al*., 2020; Said *et al*., 2021); other unspecified punishments or punitive actions (Abouchadi *et al*., 2013; Kouanda *et al*., 2022; Russell *et al*., 2022); an external investigation (Melberg *et al*., 2019); having to explain to higher officials, often politicians (Abouchadi *et al*., 2013; Kouanda *et al*., 2022); being called out or reprimanded during death review or the risk of being considered unskilled by one’s colleagues (Abouchadi *et al*., 2013; Armstrong *et al*., 2014; Shittu and Kinney, 2017); and generally feeling threatened (Kinney *et al*., 2021).

### Targets, exacerbating factors and effects of blame

Health workers were reported as the actors most often blamed in relation to the MDSR (WHO, 2016a; Abebe *et al*., 2017; Lardi *et al*., 2019; Kinney *et al*., 2020; Abouchadi *et al*., 2022). Among the category of health workers, low-level or front-line health workers and junior doctors seemed to be subjected to blame more often than others (Scott and Dairo, 2015; Singh *et al*., 2015; Melberg *et al*., 2019; Naik *et al*., 2020; Cahyanti *et al*., 2021). A study from Ethiopia reported that while doctors were concerned about formal legal accusations, lower-level health workers were worried more about informal sanctions (Melberg *et al*., 2020). Apart from health workers, health institutions (Cahyanti *et al*., 2021; Boyi *et al*., 2022), traditional birth attendants (WHO, 2014; Russell *et al*., 2022), family members of the deceased, community members and the deceased women themselves were also subjected to blame of some form (Scott and Dairo, 2015; Melberg *et al*., 2019; Willcox *et al*., 2023).

The studies also revealed factors reinforcing the sense of blame and insecurity associated with the MDSR process. Several studies reported, for instance, that the increase in malpractice cases had such an effect (Shellhaas and Conrey, 2018; Melberg *et al*., 2019; 2020). Community members in Ethiopia also hesitated to report community maternal deaths owing to the heightened emphasis placed on institutional delivery (Abebe *et al*., 2017). Other contributing factors included a strong political will to reduce maternal deaths (Abebe *et al*., 2017; Melberg *et al*., 2019; Kouanda *et al*., 2022), hostile media coverage of such deaths (Abouchadi *et al*., 2022; Kouanda *et al*., 2022) and lack of understanding about the objectives of the MDSR (Armstrong *et al*., 2014; Kinney *et al*., 2021; Russell *et al*., 2022).

Effects of blame are also commonly discussed in the studies. Accordingly, the perception that the MDSR was punitive or an understanding that actors involved were not adequately protected led to intentional non-reporting or under-reporting and in some cases selective reporting of maternal deaths (Singh *et al*., 2015; Mutsigiri-Murewanhema *et al*., 2017; Melberg *et al*., 2019; Kouanda *et al*., 2022; Yameogo *et al*., 2022). It is also observed that the fear associated with the MDSR particularly resulted in the non- or under-reporting of abortion-related maternal deaths in settings where abortion is criminalized or socially stigmatized (WHO, 2014; Singh *et al*., 2015; Naik *et al*., 2020; Russell *et al*., 2022; Yameogo *et al*., 2022). Other effects identified included discouraging health workers from participating in review meetings or when they did, hesitance to freely speak (WHO, 2014; Said *et al*., 2021; Boyi *et al*., 2022; Russell *et al*., 2022) and intentionally misclassifying, falsifying or misattributing causes of death (WHO, 2016a; Abebe *et al*., 2017; Melberg *et al*., 2019; Willcox *et al*., 2023).

Moreover, several studies indicated that fear of legal consequences associated with the MDSR directly affected access to and the quality of healthcare. Among others, health workers and health facilities were found to unnecessarily refer severely ill women. In other cases, pregnant women were refused admission and treatment due to the fear of being accused in case of maternal death (Singh *et al*., 2015; Melberg *et al*., 2020; Abouchadi *et al*., 2022; Russell *et al*., 2022). Similarly, it was reported that increased vulnerability to accusations of death deterred junior doctors from pursuing obstetrics as a career (Melberg *et al*., 2020; Willcox *et al*., 2023).

To mitigate the manifestations and effects of blame in the context of the MDSR, most studies provided certain recommendations. Among the most frequently reiterated suggestions were providing training, assuring health workers about the ‘no name, no blame’ principle and orienting health workers and managers about the objectives of the MDSR, emphasizing the importance of adhering to the non-blame principle (WHO, 2014; Mutsigiri-Murewanhema *et al*., 2017; Smith *et al*., 2017a; Compaore *et al*., 2022; Yameogo *et al*., 2022). A considerable number of studies also called for legal protection, including the adoption of appropriate laws as a potential solution, even though these calls were frequently general in their framing (Abebe *et al*., 2017; Shittu and Kinney, 2017; Kodan *et al*., 2021; Willcox *et al*., 2023).

### The use of MDSR findings for unintended purposes

This theme describes the extent to which the MDSR files and documents are safeguarded against unintended use and particularly if and how they are insulated from legal and administrative processes, as recommended by the WHO Technical Guidance (WHO, 2013) and relevant literature. We found limited papers discussing this aspect.

From the relevant studies, there were a general understanding and consequent concern that the MDSR process could be used in legal or disciplinary proceedings (Scott and Dairo, 2015; Abebe *et al*., 2017; Melberg *et al*., 2019; Cahyanti *et al*., 2021; Yameogo *et al*., 2022). Among others, MDSR-related documents were used in legal proceedings in Tanzania (Said *et al*., 2021). Local authorities also reportedly used such files to attribute fault to healthcare professionals and facilities or evaluate the performance of facility managers (Melberg *et al*., 2019; Abouchadi *et al*., 2022). Moreover, MDSR files and findings were deliberately referred to the human resources department of health facilities. They were then utilized to discipline personnel suspected of failures revealed by the process (Cahyanti *et al*., 2021; Said *et al*., 2021). Apart from the deliberate use of the MDSR for punitive purposes, several studies also documented the challenge of separating the MDSR from other punitive and disciplinary processes (Armstrong *et al*., 2014; Abouchadi *et al*., 2022; Russell *et al*., 2022; Willcox *et al*., 2023). As such, the need to ensure separation, including by law, was emphasized (Shittu and Kinney, 2017; Lardi *et al*., 2019). While a few studies indicated that the MDSR is insulated from other processes by law, for instance, in South Africa (Smith *et al*., 2017a), other studies reported that in some countries, it is prohibited to use the MDSR in legal or disciplinary measures, without clarifying how this is enforced (Willcox *et al*., 2023).

### Legal regulation of the MDSR

Under this theme, we present findings related to the extent to which laws, serving as regulatory and facilitative tools for the MDSR or its components, were discussed in the reviewed studies.

#### Means of regulating the MDSR

The literature demonstrated that legislation specific to the MDSR is rare. Only one study from Chad discussed a specific law and its provisions governing many aspects of the MDSR (Kouanda *et al*., 2022). Other studies referenced ministerial orders, decrees and circulars as tools for governing the respective MDSR systems without, however, providing details about their content (Agaro *et al*., 2016; Cahyanti *et al*., 2021; Kouanda *et al*., 2022). It appears that these instruments mainly governed certain components of the MDSR cycle. For instance, in Cameroon, it is reported that the notification and the formation of review committees were mandated by a ministerial decree (Bongajum *et al*., 2021). Similarly, in Benin, it is noted that separate laws regulated notification and the establishment of MDSR committees (Boyi *et al*., 2022). In the absence of more formal laws, other quasi-legal instruments, including audit charters, guidelines, codes of conduct and memoranda of understanding, were used to govern or facilitate aspects of the MDSR (Shittu and Kinney, 2017; Smith *et al*., 2017b; Kinney *et al*., 2020; Willcox *et al*., 2023).

The existence of laws or legal frameworks was discussed as a strength of the MDSR system (Goodman *et al*., 2013; Kodan *et al*., 2021). However, the studies highlighted a notable lack of sufficient laws, which was linked to weak implementation in many countries (Kinney *et al*., 2021; Abouchadi *et al*., 2022; Willcox *et al*., 2023). Calls were thus made for appropriate laws to clarify the mandates and obligations of diverse actors, offer essential protection for these actors and more clearly outline the objectives of the MDSR (Armstrong *et al*., 2014; Abouchadi *et al*., 2022; Compaore *et al*., 2022). However, discussions rarely extended beyond the need for laws or legal protection. Moreover, in countries where laws on certain aspects of the MDSR were partially available, non-compliance continued as a challenge (Scott and Dairo, 2015; Bongajum *et al*., 2021; Kouanda *et al*., 2022).

#### Mandatory reporting of maternal deaths

Among the legal interventions that can support the MDSR, the imposition of mandatory reporting or notification was widely reported in the studies. Accordingly, in most of the countries studied, maternal death was a notifiable event. While some studies indicated that notification was mandated by law (WHO, 2014; Scott and Dairo, 2015; Shellhaas and Conrey, 2018), the majority did not clarify how this was effected. It is also indicated that mandatory notification could be introduced at various stages: before the adoption of the MDSR system (Abouchadi *et al*., 2022), alongside its introduction (Abebe *et al*., 2017) or as an afterthought, informed by lessons from the review system (Goodman *et al*., 2013). Several studies highlighted that notification was implemented by adding maternal death to the list of other reportable diseases and health conditions and by incorporating it into existing health information systems (Abebe *et al*., 2017; Boyi *et al*., 2022; Kouanda *et al*., 2022).

## Discussion

This scoping review shows that a modest but growing body of literature touches upon different legal aspects of the MDSR system. African countries are predominantly featured in the studies, likely due to the widespread adoption of the system there in response to high maternal mortality rates and inadequate civil registration systems. A major legal problem affecting the programme’s implementation highlighted in the review is the fear of blame. This primarily arises from inadequate legal protection and is further reinforced by the portrayal of the MDSR as an accountability mechanism focused predominantly on punitive measures. The review underscores the need for a strong legal framework to support effective MDSR implementation. Key aspects of this framework include facilitating the notification of maternal deaths, ensuring access to health information systems while maintaining the privacy of data, protecting participants—especially health workers—from legal repercussions and establishing and authorizing review committees properly. To elucidate the nature of the legal issues and concerns identified, we discuss the findings according to major themes that emerged in the review.

### Law and its neglected role in the regulation of the MDSR

The scoping review highlights the limited attention given to the legal dimension of the MDSR, in both coverage and the language used. For instance, even though the MDSR involves the exercise of public power, including the authorization of state organs to collect and access health information, this aspect was rarely raised. Consequently, the studies generally depict the MDSR as a mere technical tool that does not require a legal basis to exist. Similarly, while discussions about the safety of data abound in the studies, no attempt is made to articulate them in terms of the right to privacy, the closest legal expression of the interests discussed. Furthermore, legal tools such as the right to access to information or court subpoena of witnesses, which are common themes in the public health surveillance legal literature (Hodge, 2003; Neslund, 2007; Neslund *et al*., 2010; Gostin and Wiley, 2016), are not mentioned in the studies. A similar observation can be made regarding mandatory reporting, one of the widely reported themes in the review. The studies do not discuss the extent to which the relevant laws on mandatory reporting fit the purposes and fast-paced process of the MDSR.

Law exerts a powerful influence on health by structuring, perpetuating and mediating risk factors, underlying conditions and facilitating interventions (Burris *et al*., 2010; Gostin *et al*., 2019). This foundational role of law in shaping public health outcomes underscores the significance of legal frameworks in supporting health systems That said, the limited attention paid to laws and the legal dimension of the MDSR in the studies can be attributed to various factors. To begin with, the role of law in governing public health or framing issues in public health as legal has traditionally been nominal (Fox, 2001; Institute of Medicine, Board on Population, Health, 2011; Gostin *et al*., 2019). This is partly explained by a tendency among health professionals, who mostly oversee public health programmes, to prefer technical and more flexible approaches to governing public health work and resist formal and often power-restraining laws (London, 2008; Gostin and Wiley, 2016). Furthermore, laws represent just one of many regulatory tools available to health administrators. We also posit that the predominance of researchers with medical or health science backgrounds in the studies included for this review has likely played a role in the limited attention given to legal issues. It also appears that legal scholars have not been engaged in studying the MDSR. Future studies on MDSR implementation may benefit if they pay increased attention to the legal issues that affect its functioning. In the same vein, it is imperative to include legal competence in both research and MDSR supervision teams.

### Informational privacy as a central concern

The review demonstrates that data used in MDSR contain information that identifies individuals. In many cases, collected data seem to be sufficient to potentially identify the deceased, relatives and health workers who provided care, among others. Even though mitigating measures such as anonymizing identifiable data were in some instances required, they appear to be insufficient. To begin with, these requirements come late in the MDSR process (Scott and Dairo, 2015; Smith *et al*., 2017a; Abouchadi *et al*., 2022). Moreover, it is unclear whether anonymization, when implemented, is mandated by law and whether specific anonymity standards are provided in the respective countries. Consequently, insufficient anonymity remains a major challenge for the MDSR, feeding and reinforcing the fear of unwarranted blame, mainly among health workers (Armstrong *et al*., 2014; Kinney *et al*., 2020; Abouchadi *et al*., 2022; Willcox *et al*., 2023).

Informational privacy is at stake here. As a right recognized under international and national laws, informational privacy guarantees rights holders to control the acquisition, use or disclosure of their identifiable data. As such, obtaining consent from the right holder is generally necessary to legitimize the collection, use, transfer or other forms of processing their data (Bygrave, 1998; Neslund, 2007; Witzleb, 2014; Ball, 2017; Johnson, 2018; Mariner *et al*., 2019). Like many other rights, however, the right provides exceptions under which personal data can be processed without the consent of the right holder under such conditions as legality, necessity and proportionality. Briefly stated, a law authorizing the exception is necessary. Besides, it should be demonstrated that the data are essential for the intended purpose and that the impact on the right holder is proportionate to the benefits gained. It is also part of the right to have personal data effectively de-identified once it achieves the purpose for which it was given or acquired (Bygrave, 1998; Hodge, 2003; Witzleb, 2014; Ball, 2017; Mariner *et al*., 2019; Schneider, 2019).

Closely related to informational privacy is the protection of confidentiality. Often considered a subset of the right to privacy, confidentiality represents the safeguarding, primarily from disclosure, of information shared within relationships of trust and confidence. While informational privacy grants individuals the right to restrict access to information that is legally recognized as private or personal, its counterpart, confidentiality, emphasises the responsibility of those entrusted with confidential information not to disclose it to others or use it in a manner that contradicts its intended purpose (Lowrance, 2012; Gostin and Wiley, 2016; Hulkower *et al*., 2020).

Public health is a legitimate ground for which an exception is made under the conditions outlined. As such, for personal data to be used without the informed consent of the right holder, this needs to be mandated by law, and the necessity and proportionality requirements should be met. In the context of the MDSR, this implies that a national law should mandate it, specifying the manner in which identifiable data can be processed. Efforts to improve MDSR implementation need to give due attention to these requirements, as a matter of legal obligation. The availability of identifiable data concerning health workers in files utilized for death review, along with the confidential nature of the MDSR process, underscores the critical need to ensure privacy and uphold strict confidentiality, in compliance with legal obligations.

This is also justified on instrumental grounds. It is argued that people cooperate better in providing health data, which forms the backbone of public health works, if they trust that their data are collected, used and shared responsibly (Hodge, 2003; Gostin and Wiley, 2016). This can arguably be extended to health workers and their participation in the MDSR. By providing rules and procedures for weighing competing interests, laws on privacy rights and their limitations have the potential to improve trust in the system. This aligns with the growing understanding that health programmes are more effectively implemented when the rights and values they implicate are duly taken into account and carefully balanced (Gostin and Mann, 1994; Bygrave, 1998; Toebes, 2015; Annas and Mariner, 2016). From the review, it is evident that the mere inclusion of principles in technical guidance are insufficient for ensuring data privacy and process confidentiality within the MDSR framework. Given that privacy is a well-established interest and extensive jurisprudence exists to balance rights in a manner that advances public health, implementing countries are advised to consider legislative measures to overcome these inadequacies and support the effective implementation of the health programme.

### Inadequate protection for health workers

The review documents health workers’ continued worries about the MDSR system (WHO, 2016a; Shittu and Kinney, 2017; Melberg *et al*., 2019; Boyi *et al*., 2022; Willcox *et al*., 2023). Factors that reinforce the fear of blame around the MDSR range from heightened political importance attached to mortality numbers in global health to the lack of sufficient legal protection that the objectives and confidential nature of the MDSR necessitate (Abebe *et al*., 2017; Melberg *et al*., 2020; Kouanda *et al*., 2022). The politicization of health data and its potential to compromise the integrity of health systems and accountability mechanisms are well documented (Adams, 2016; Østebø *et al*., 2018; Blouin, 2020; Davis, 2020). The review also highlighted that the fear of blame not only hinders MDSR implementation but also may lead to additional negative health outcomes. For instance, there are indications that some health workers, feeling insecure about the requirement to register and review maternal deaths, refer severely ill women to other facilities unnecessarily or decline to provide care.

To support regulatory efforts to improve the MDSR, we recommend a broad classification of the effects of blame on MDSR into two categories: legal and non-legal. Associations of the MDSR with litigations (Melberg *et al*., 2019; Kinney *et al*., 2021; Mukinda *et al*., 2021), suspension from work (Kinney *et al*., 2020; Mukinda *et al*., 2021), other disciplinary actions (Shittu and Kinney, 2017; Mukinda *et al*., 2021) and oral or written warnings (Shittu and Kinney, 2017; Said *et al*., 2021) seem to stem from inadequate legal frameworks protecting health workers in relation to their involvement in the MDSR. These outcomes often involve or seem to involve legal institutions and pertain to rights and interests frequently protected by laws, such as liberty, job security or employment benefits, which broadly qualifies them as legal.

Legislative initiatives seem to have the potential to mitigate these challenges. While the particular legal design adopted depends on the legal system in question, laws such as the laws of evidence, procedure and labour laws deserve particular attention, considering their strong bearing on the legal forms of blame. Examining the extent to which existing laws address the challenges that the MDSR is facing should form part of the solution. This is necessary because merely declaring the separation of the MDSR from legal or administrative measures, expressing a political commitment to ensure privacy and confidentiality or adhering to the ‘no blame’ approach is unlikely to have a legal effect when concrete legal cases arise. Public health law studies document that the quality of such ‘incidental laws’ substantially affects the efficacy of health programmes (Burris *et al*., 2010; Gostin and Wiley, 2016). Related to this, it is also important to strengthen dispute settlement and complaint-handling mechanisms in the health system. Without robust procedures, interested parties tend to resort to using the MDSR as a forum for litigation and blame (Melberg *et al*., 2020; Said *et al*., 2021).

The studies also report other forms of blame that are less directly related to legal processes or institutions, falling broadly under the ‘non-legal’ category. For instance, health workers are reprimanded or are made to feel that they are judged as unskilled during death reviews (Armstrong *et al*., 2014; Shittu and Kinney, 2017; Melberg *et al*., 2019) or that after the MDSR, they need to explain results to higher officials, often politicians (Abouchadi *et al*., 2022; Kouanda *et al*., 2022). Clarifying the objectives and importance of separating the MDSR from punitive and individual evaluation systems through training and appropriate reminders appears to be an important way to deal with these challenges. Persuasion and public awareness are considered important tools for implementing public health policies (Gostin and Wiley, 2016). Integrating legal solutions with non-legal interventions, based on the nature of the blame and the legal system involved, can potentially enhance MDSR implementation.

### MDSR and accountability: misunderstandings

In connection with the fear of blame, the language of accountability recurs in the studies reviewed. In this regard, we observed diverse and at times conflicting usages of the notion. Generally, accountability in the context of the MDSR is portrayed as both an ally and a foe and is discussed at two main layers: the health system level and the individual level.

The studies typically portray a positive correlation between MDSR and accountability when the focus is on the health system. As a value or culture in the health system within which the MDSR functions, it is reported that accountability supports the MDSR or is produced or enhanced by it. Here, accountability relates to transparency and better coordination in the health system, its willingness and ability to assess actions and learn, explain the circumstance of death to bereaved families (Willcox *et al*., 2023) and most importantly act based on recommendations and ensure effective follow-up (Compaore *et al*., 2022; Kouanda *et al*., 2022; Willcox *et al*., 2023). Overall, the MDSR serves as an accountability mechanism—a tool designed to enhance maternal health through monitoring, reviewing and responding.

On the other hand, when used in relation to individual actors in the MDSR, accountability mostly represented blameworthiness, punishment or other negative consequences, resulting from some form of failure. Accountability thus embodies something workers want to avoid and as such a challenge to MDSR implementation (Shittu and Kinney, 2017; Melberg *et al*., 2019; Said *et al*., 2021). The MDSR is thus seen in this context as a forum and vehicle to identify those responsible for maternal death and punish their actions.

The dual use of the term ‘accountability’ is understandable, given its complexity. Accountability is a layered concept involving transparency, taking responsibility, identifying those responsible and sanctioning inappropriate action (Schedler *et al*., 1999; Brinkerhoff, 2004).

This conflicting understanding may be exemplified by the challenging roles of directors or facility managers in the MDSR. While many of the studies report that chief medical directors chair MDSR reviews (WHO, 2014; Shittu and Kinney, 2017; Mukinda *et al*., 2021), this position is not always appreciated. While the involvement of such officers in death reviews is considered crucial to conducting orderly meetings and translating recommendations into action, their responsibility to ensure quality health services delivery and their ability to take administrative measures against employees cause tensions in meetings (Said *et al*., 2021). Both roles are often described as accountability. A similar dilemma is observed concerning the involvement of lay members of the community in reviews—while their participation in the process helps with feedback, it also heightens suspicion among health workers to prevent them from speaking freely in the presence of ‘outsiders’ (WHO, 2014; Ayele *et al*., 2019).

Some observations can be drawn from the discussion on accountability. Notably, the distinction underscores that the primary purpose of the MDSR is to identify and address failures within the healthcare system. Moreover, utilizing the MDSR for punitive actions or disciplining individual implementers is counterproductive. It is evident that maintaining a completely blame-free process is challenging, and the argument is not against individual responsibility for illegal or unprofessional conduct. Rather, it emphasizes that the MDSR is not a suitable platform for regulating healthcare workers. This necessitates a careful delineation of the process from punitive measures, without implying that professional misconduct should be tolerated.

## Limitation

This research is subject to certain limitations that should be considered when interpreting the findings. The definition of ‘legal issues’ presents the first challenge. ‘Legal issues’ are defined in reference to laws. However, there is no universal consensus on what topics and interests should be regulated by law. The decision to regulate, for instance, a health programme by law depends on multiple factors and considerations in the country concerned (Coker and Martin, 2006). This renders the scope of ‘legal issues’ inherently indeterminate. This methodological challenge led to a circular process of literature selection and analysis: starting with a broad definition of what is considered a ‘legal issue’ and working with categories drawn from conventional understandings of the expression in the context of the MDSR. This similarly reduces the utility of the proposed distinction between legal and non-legal forms of blame. Another limitation is the variability of terminologies used to describe the MDSR in the literature. This might have led to the exclusion of some relevant materials. The limited discussion on legal issues in the studies also weakens the robustness of our observations and conclusions. This scoping review, therefore, is limited to providing illustrations rather than definitive conclusions about the legal aspects of the MDSR. The potential bias introduced by the authors’ backgrounds represents another limitation. The first author’s legal education and the last author’s extensive work with the MDSR may have influenced the selection and review of the studies. However, we trust that the diverse disciplinary backgrounds of the authors and their varied experiences with the MDSR, coupled with regular meetings to discuss the literature, helped to mitigate this bias. Lastly, it is important to note that, unlike systematic reviews, our research did not involve an in-depth quality assessment of the included studies or an evaluation of the risk of bias. This reflects the exploratory and broad-ranging nature of our research approach.

Despite these limitations, the study’s strengths—particularly its adherence to recommended methodological steps and considerations, its enhanced conceptual framework and the diligent inclusion of literature with pertinent data—ensure that the findings offer valuable insights.

## Conclusion

To our knowledge, this review represents the first attempt to empirically map the legal dimension of the MDSR, using an enhanced conceptual framework. It shows that the relevant literature mostly touches on some legal issues at a surface level. These studies highlight the importance of providing sufficient legal frameworks for the MDSR to function effectively and point out that existing laws in many countries are inadequate. Particular attention is placed on insufficient protection of privacy and confidentiality as well as inadequate insulation of the MDSR process from being used for punitive purposes. Accountability also emerges in the studies as both an objective of the MDSR and synonymous with sanctions flowing from the MDSR. These challenges foster a blame culture that negatively affects implementation at all stages of the MDSR cycle. This potentially compromises the MDSR’s core objectives of informing corrective actions and monitoring progress towards improved maternal health.

This review addresses a significant gap in the literature concerning the legal dimensions of the MDSR, a critically overlooked area. By elucidating themes and subject matters that make up the regulatory environment of the MDSR, it broadens understanding of the requisite legal framework for its effectiveness. The enhancements we propose to the conceptual framework originally developed by Bain and Kongnyuy provide a robust analytical tool for future research. This work also enriches the sparse literature on the role of law in public health. More importantly, the review supports ongoing efforts, particularly by the WHO, to strengthen the health programme as a vital component of the health system to improve maternal health. It achieves this by highlighting the current state of knowledge in the area and identifying gaps that require prior and further attention. Given that the MDSR system is now being expanded to encompass maternal morbidity and perinatal deaths, the urgency for these improvements cannot be overstated. Moreover, improved data production and utilization, supported by necessary legal frameworks, not only enhance decision-making but also empower many low- and middle-income countries to progressively produce their own vital health and development data, reducing current reliance on global estimates.

## Supplementary Material

czae071_Supp

## Data Availability

Additional data underlying this review can be shared upon reasonable request to the corresponding author.

## Footnotes

1. MATRISET, the organizer of the seminar, is a project that studies the implementation of the MDSR system in Ethiopia and Tanzania. More about the project is available at https://www.uib.no/en/cih/155099/facilitating-%C2%ABreal-life%C2%BB-implementation-maternal-death-reporting-and-review (accessed on 25 July 2024)

2. adj2 = word 2 is the next word or the maximum second (2) after word 1.

3. For definitions of these terms, see Scott and Dairo (2015), Kinney *et al*. (2020) and WHO (2004, 2013, 2016a).

## Reflexivity statement

The authorship demonstrates a balanced representation in terms of gender, seniority and regional location. Notably, the first author, M.B.K. (male), is a junior researcher from Ethiopia. A.M. (female), a mid-level researcher, is based in Norway. Both K.M.M. (female) and I.M. (female), are senior scientists based in Norway with a research focus on Africa.
