# Supplementary material for: Legal issues in the implementation of Maternal Death Surveillance and Response: a scoping review
Source: Health Policy Plan. 2024 Aug 3;39(9):985–99. doi: 10.1093/heapol/czae071 (PMC11474607; doi:10.1093/heapol/czae071)
Supplement: czae071_Supp [file czae071_supp.zip › Supplementary File 1_Review Protocol.doc]

**Supplementary File 1:** Review protocol

Title: Legal issues arising in the implementation of Maternal Death Surveillance and Response (MDSR): a scoping review protocol

Date: November 2022

# 1. Preliminary notes

This protocol guides a literature review and analysis intended for publication as an academic journal article, forming part of a PhD project. Review protocols provide a roadmap for reviewers regarding the direction and conduct of the review, and their popularity is increasing (Peters *et al.* 2022). This protocol is developed based on the well-established Arksey and O’Malley approach for scoping reviews, as further developed by others (Arksey and O'Malley 2005, Daudt *et al.* 2013, Aromataris 2020). The Arksey and O’Malley approach consists of six stages: (1) identifying the research question, (2) identifying relevant studies, (3) study selection, (4) charting of data, (5) collating, summarizing, and reporting the results, and (6) optional consultations. These stages are outlined in the method section of this protocol.

It is common for scoping review authors to deviate from protocols as they increase their awareness of the process (Peters *et al.* 2022). Hence, some adjustments to this protocol may be introduced in the course of the review, in which case these changes will be reported in the resulting paper.

# 2. Administrative information

2.1. *Registration/publication of the protocol*

The protocol will be published as a supplement to the review paper, and thus also the PhD thesis which it will form a part of.

2.2. *Authors of the protocol*

This protocol is written by Mulu Beyene Kidanemariam, Ingrid Miljeteig, and Karen Marie Moland.

2.3. *Support sources*

The research is carried out as part of a PhD project at the Faculty of Law, University of Bergen, Norway.

# 3. The rationale of the review

The Maternal Death Surveillance and Response (MDSR) is a health programme designed to help countries with poor civil registration systems better appreciate the scale of maternal mortality in their respective jurisdictions, understand the causes of such deaths, and thereby take corrective measures to improve maternal health care. Introduced in 2013, it is now being adopted in a considerable number of countries (WHO 2013, WHO 2023). The MDSR contains distinct activities to identify, report, and review all maternal deaths; establish causes and assess the preventability of death; and, based on the data gathered, respond to failures identified to prevent similar occurrences (Abebe *et al.* 2017, Smith *et al.* 2017). The cycle is often divided into four stages, namely, identification and reporting, death review, analysis and recommendation, and response and follow-up. In this process, relevant information is gathered from such sources as family members, community members, patient records, and healthcare workers involved in caring for the deceased woman in question. With a view to understanding the circumstances of the death, the MDSR process requires open discussion among reviewers.

Considering the investigative nature of the MDSR and the involvement of various actors in the process, its implementation is often associated with certain legal issues and concerns. Among others, a great deal of MDSR activities touch upon some legally protected rights, including informational privacy rights. The establishment of causes and preventability of deaths also heightens concerns of responsibility, mainly among health workers. Legal issues also arise concerning the legal authority to undertake the programme. This is so, as the MDSR involves using a great deal of state power such as obligatory notification of death, and the use and transfer of data (WHO 2016, Bain and Kongnyuy 2018). In light of these considerations, the WHO MDSR Technical Guidance, which serves as the primary regulatory framework for the intervention, advises MDSR-implementing countries to put in place appropriate legal frameworks, including necessary protections for those involved in the process (WHO 2013).

Research on the implementation of the MDSR reveals various challenges. Notably, the lack of an appropriate legal framework and insufficient legal protection for health workers are significant obstacles. Such deficiency contributes to the weak implementation of the programme. (WHO 2014, Agaro *et al.* 2016, WHO 2016, Smith *et al.* 2017, Ayele *et al.* 2019, Melberg *et al.* 2019, Tura *et al.* 2020, Kinney *et al.* 2021). However, the nature, forms, and manifestations of these legal issues and concerns are not explored in the literature, and the dearth of work in this regard is particularly noted (Smith *et al.* 2017, Bain and Kongnyuy 2018, Kinney *et al.* 2021). A preliminary search we conducted on PubMed and Google Scholar also confirms this. The need to map the legal terrain of the MDSR thus emerges as a necessary initial step to explore the role law can play in its improvement.

# 4. Study objectives

The scoping review aims to map and report the available literature on the nature and forms of legal topics and issues that arise in the implementation of the MDSR. Additionally, we will also explore and visualize the overall research activity on the subject.

# 5. Research Method

## *Scoping review design*

A scoping review aims to “… map the literature on a particular topic or research area and provide an opportunity to identify key concepts; gaps in the research; and types and sources of evidence to inform practice, policymaking, and research” (Daudt *et al.* 2013). Scoping reviews apply a transparent data generation and analysis, and focus “around priorities for research, clarifying concepts and definitions, providing research frameworks or providing background, or contextual information on phenomena or concepts” (Arksey and O'Malley 2005). Moreover, the research design is relevant to examine “emerging evidence when it is still unclear what other, more specific questions can be posed and valuably addressed” (Armstrong *et al.* 2011). Furthermore, it has the advantage of catering to broadly framed question and is recommended to examine the extent, range, and nature of research activity and identify research gaps in the existing literature, particularly where no research has been conducted and the review is exploratory (Arksey and O'Malley 2005, Pham *et al.* 2014, Tricco *et al.* 2016, Munn *et al.* 2018). A scoping review is used as an alternative to a systematic review, which addresses a particular question when significant comparable evidence is available (Munn *et al.* 2018).

The broad scope of the research objective, coupled with the apparent lack of relevant works on the topic, makes a scoping review a suitable research design for this work. Below, we outline how we intend to conduct the scoping review, following the six stages that Arksey and O’Malley (2005) developed.

## ***Stage 1: Identifying the research question***

The main question for the scoping review will be: What is known from existing literature about legal issues that arise during the implementation of the Maternal Death Surveillance and Response? To properly manage questions, Aksey and O’Malley suggest that reviewers should limit the parameters of the research question by, among others, defining ambiguous terms included in the question. (Arksey and O'Malley 2005) Accordingly, the expression ‘legal issues,’ which is a central element of the question, requires clarification. Black’s Law Dictionary defines the term ‘legal’ as follows: ‘of or relating to law; falling within the providence of law,’ ‘established, required, or permitted by law’, and ‘of or relating to law as opposed to equity’ (Black and Garner 2004). Hence, legal issues are understood broadly, encompassing the first three elements of the definition.

In the context of the MDSR, these would include aspects of the MDSR process covered by existing legislative tools or judicial decisions; or are envisaged to be regulated by law, for instance under the establishing technical guidance; or are likely to or do give rise to tensions or concerns that conventionally require the application of law or involvement of judicial or semi-judicial organs for their regulation in the relevant jurisdiction. The law in question does not need to be particularly enacted to regulate the MDSR and may come in various forms.

## ***Stage 2: Identifying relevant studies***

To address broader questions, scoping reviews need to be as comprehensive as possible, allowing the inclusion of any literature as evidence sources. (Arksey and O'Malley 2005, Peters *et al.* 2022). However, feasibility considerations, including time and resources, can also be accommodated (Levac *et al.* 2010). Pilot testing and consulting experts are also recommended (Arksey and O'Malley 2005, Spencer and Eldredge 2018).

Sources for our search will be identified following preliminary pilot testing and consultations with librarians at…. Accordingly, suitable databases, search engines, and particularly relevant websites will be searched. Among others, Medline, Embase, Web of Science, PubMed Central, JSTOR, HeinOnline, West Law, and … databases, Google Scholar, as well as the WHO library will be considered. To identify additional relevant materials not yet captured, the reference list of selected publications will be manually searched (Pham *et al.* 2014). The combined use of databases, open search engines, the WHO website, and manual search of references enables us to screen not only journal articles but also grey literature relevant to the study.

## ***Stage 3: Study selection***

#### 3.1. Citation management

All citations will be imported into EndNote20 citation manager, and duplications will be removed through the system.

#### 3.2. Inclusion and exclusion criteria

We will consider all materials, published in English since 2010 that discuss legal issues arising during the implementation of the MDSR. The year 2010 marks the start of the policy initiative that ultimately established the MDSR (Danel *et al.* 2011). We chose to focus on publications dealing with MDSR implementation to better understand the empirical experiences of countries. Our observation from the literature also indicates that the research question is likely to be discussed in publications that pertain to the implementation of the MDSR. The publications to be included may discuss maternal death review alone or alongside perinatal death review and response systems. However, those exclusively dealing with perinatal/neonatal review systems or confidential enquiries into maternal death will be excluded, as they differ from MDSR in either their focus or process (WHO 2004, Moodley *et al.* 2014).

#### 3.3. Screening process

We will ensure that titles and abstracts will be at least screened by one reviewer and verified by another (Aromataris 2020). We will also regularly meet to discuss and possibly refine selection criteria, as recommended (Levac *et al.* 2010). Full text will also be conducted either by two reviewers who will review independently or by one reviewer to be verified by another reviewer. We will also pilot-test the screening (Peters *et al.* 2022). Discrepancies between and among reviewers will be settled through discussion.

## ***Stage 4: Charting data***

Charting in a scoping review is a “technique for synthesizing and interpreting qualitative data by sifting, charting and sorting material according to key issues and themes…” (Ritchie and Spencer 1994). The research team will develop a charting form through discussion. One author will conduct a pilot test on selected publications, and at least another reviewer will verify the data, as recommended (Aromataris 2020). Considering reported challenges in devising a charting form that caters to evolving data and the likelihood of lacking enough information on all agreed fields (Arksey and O'Malley 2005, Levac *et al.* 2010), we will be open to continually updating the charting form through discussion. We will chart study characteristics, including author(s), year of publication, country or countries of focus, level of study, research design, as well as relevant research results.

## ***Stage 5: Collating, summarizing, and reporting the results***

This stage aims to present a narrative account of existing literature, and for this, an analytical framework or thematic construction is needed. Authors are also supposed to devise ways to best present an overview of all materials reviewed, which may change and evolve (Arksey and O'Malley 2005, Peters *et al.* 2022). We also note that at a protocol level, it is considered sufficient to provide some indication that the authors considered how to present anticipated results (Peters *et al.* 2022).

We are weighing up three possibilities for organizing the findings. The first is to follow the MDSR cycle (WHO 2016) and try to identify legal issues in each stage. This approach seems to allow us to capture many of the potential legal issues. However, it may not accommodate some apparent legal issues, including issues of legal authority to conduct MDSR or health workers protection, common themes in the MDSR literature. Overlap among the MDSR stages may also be prominent. The second option is to use a categorization developed by Bain and Kongnyuy (2018). It identifies three categories under which legal issues in MDSR can be summarized, namely data, persons, and use of findings. While this formulation appears relatively more systematic, it might not accommodate more general topics.

The third possibility we are considering using is a recently developed Conceptual Implementation Framework for M/PDSR (Kinney *et al.* 2019). The framework charts MDSR implementation factors under four domains, namely the way the intervention is structured (intervention); the motivation and skill of individuals involved (individual); organizational culture, implementation climate, and coordination within the programme (inner setting); and factors external to the programme factors, such as policy and funding (outer setting). The wide reach of the domains, while capable of accommodating a vast array of legal issues, might prove too broad to guide the charting process in our review, considering the scarcity of material on the topic. We will examine the material we can find in the relevant publications against each alternative. We are also open to inductively constructing a new analytical framework if that would help us better present and analyze findings.

The review decision process will be reported using an adapted ‘Preferred Reporting Items for Systematic Reviews and Meta-Analyses’ extension for scoping reviews flow diagram (Tricco *et al.* 2018)

## ***Stage 6: Consultations***

This protocol and preliminary findings will be presented in relevant forums to generate ideas for improving the review and disseminating the findings.

# 6. References

Abebe B *et al.* 2017. 'We Identify, Discuss, Act and Promise to Prevent Similar Deaths': A qualitative study of Ethiopia's Maternal Death Surveillance and Response system. *BMJ Global Health* **2**(2): e000199.

Agaro C *et al.* 2016. The conduct of maternal and perinatal death reviews in Oyam District, Uganda: A descriptive cross-sectional study. *BMC Women's Health* **16**: 38.

Arksey H, O'Malley L. 2005. Scoping studies: Towards a methodological framework. *International Journal of Social Research Methodology* **8**(1), 19-32.

Armstrong CE *et al.* 2014. Strengths and weaknesses in the implementation of Maternal and Perinatal Death Reviews in Tanzania: Perceptions, processes and practice. *Tropical Medicine & International Health* **19**(9): 1087-95.

Aromataris EMZ. (eds.). 2020. *JBI Manual for Evidence Synthesis*. JBI, 2020. [https://synthesismanual.jbi.global](https://synthesismanual.jbi.global/). [https://doi.org/10.46658/JBIMES-20-01](https://doi.org/10.46658/JBIMES-20-01 (13) (20 November 2023)

Ayele B *et al.* 2019. Maternal and Perinatal Death Surveillance and Response in Ethiopia: Achievements, challenges and prospects. *PLoS One* **14**(10): e0223540.

Bain LE, Kongnyuy EJ. 2018. Legal and ethical considerations during Maternal Death Surveillance and Response. *African Journal of Reproductive Health* **22**(2): 17-25.

Black, HC, Garner BA. 2004. *Black's Law Dictionary* (8th edn.). St. Paul, Minn: Thomson/West.

Danel I, Wendy JG, Boerma T. 2011. Maternal Death Surveillance and Response. *Bull World Health Organ* **89**(11): 779-79A.

Daudt HML *et al.* 2013. Enhancing the scoping study methodology: A large, inter-professional team's experience with Arksey and O'Malley's framework. *BMC Med Res Methodol* **13**(1): 48-48.

Kinney MV *et al.* 2019. Implementation of maternal and perinatal death reviews: A scoping review protocol. *BMJ Open* **9**(11): e031328.

Kinney MV *et al.* 2021. Maternal and Perinatal Death Surveillance and Response in Low- and Middle-Income Countries: A scoping review of implementation factors. *Health Policy & Planning* **36**(6): 955-73.

Levac D *et al.* 2010. Scoping studies: advancing the methodology. *Implement Sci* **5**(1): 69-69.

Melberg A *et al.* 2019. 'Maternal deaths should simply be 0': Politicization of Maternal Death Reporting and Review processes in Ethiopia. *Health Policy & Planning* **34**(7): 492-98.

Moodley J *et al.* 2014. The confidential enquiry into maternal deaths in South Africa: A case study. *BJOG* **121**(s4): 53-60.

Munn Z *et al.* 2018. Systematic review or scoping review? Guidance for authors when choosing between a systematic or scoping review approach. *BMC Med Res Methodol* **18**(1): 143.

Peters MDJ *et al.* 2022. Best practice guidance and reporting items for the development of scoping review protocols. *JBI Evid Synth*, **20**(4), 953-68.

Pham MT *et al.* 2014. A scoping review of scoping reviews: Advancing the approach and enhancing the consistency. *Res Synth Methods* **5**(4): 371-385.

Ritchie J, Spencer l. 1994. *Qualitative Data Analysis for Applied Policy Research*. The Qualitative Researcher's Companion. A. B. a. R. G. Burgess, Routledge: 187-208.

Smith H *et al.* 2017a. Implementing Maternal Death Surveillance and Response: A review of lessons from country case studies. *BMC Pregnancy & Childbirth* **17**(1): 233.

Smith H. *et al.* 2017b. Implementing Maternal Death Surveillance and Response in Kenya: Incremental progress and lessons learned. *Global Health Science & Practice* **5**(3): 345-54.

Spencer AJ, Eldredge JD 2018. Roles for librarians in systematic reviews: A scoping review. *J Med Libr Assoc* **106**(1): 46-56.

Tricco AC *et al.* 2016. A scoping review on the conduct and reporting of scoping reviews. *BMC Med Res Methodol* **16**(15): 15-15.

Tricco AC *et al.* 2018. PRISMA Extension for Scoping Reviews (PRISMA-ScR): Checklist and explanation. *Ann Intern Med* **169**(7): 467-73.

Tura AK *et al.* 2020. Beyond No Blame: Practical challenges of conducting Maternal and Perinatal Death Reviews in Eastern Ethiopia. *Glob Health Sci Pract* **8**(2): 150-154.

WHO. 2004. *Beyond the Numbers: Reviewing Maternal Deaths and Complications to Make Pregnancy Safer*: World Health Organization.

WHO. 2013. *Maternal Death Surveillance and Response: Technical Guidance Information for Action to Prevent Maternal Death*: World Health Organization.

WHO. 2014. *Maternal Death Review in Selected Countries of South-East Asia Region*: World Health Organization.

WHO. 2016. *Time to Respond: A Report on the Global Implementation of Maternal Death Surveillance and Response*. World Health Organization.

WHO. 2023. *Maternal and Perinatal Death Surveillance and Response: Overview*, <https://www.who.int/teams/maternal-newborn-child-adolescent-health-and-ageing/maternal-health/maternal-and-perinatal-death-surveillance-and-response> (26 November 2023).
