# Supplementary material for: Legal issues in the implementation of Maternal Death Surveillance and Response: a scoping review
Source: Health Policy Plan. 2024 Aug 3;39(9):985–99. doi: 10.1093/heapol/czae071 (PMC11474607; doi:10.1093/heapol/czae071)
Supplement: czae071_Supp [file czae071_supp.zip › Supplementary File 2_Search history.doc]

**Supplementary File 2:** Literature search history

Search date: 1 December 2022

1. **MEDLINE (Ovid)**

Ovid MEDLINE(R) and Epub Ahead of Print, In-Process, In-Data-Review & Other Non-Indexed Citations and Daily <1946 to November 30, 2022>

1             ("Maternal Death Surveillance and Response" or MDSR or "Maternal and perinatal Death Surveillance and Response" or MPDSR).ti,ab,kf.  63

2             ((maternal death or perinatal death or neonatal death) adj2 (surveillance or response or review or reporting or audit)).ti,ab,kf.           253

3             1 or 2    260

4             limit 3 to yr="2010 -Current"     214

Link: <https://ovidsp.ovid.com/ovidweb.cgi?T=JS&NEWS=N&PAGE=main&SHAREDSEARCHID=1Cgn8RGtwB79AyabwLn3SHVa4ju12VAEAqy33KCZpWBTl98a4G5o5vto0Z5NGDIpH>

Comments:

adj = adjacent, meaning that search words are close to each other

adj2 = word 2 is the next word or the maximum second (2) after word 1

Ti,ab,kf. = search words must be in either title, abstract or authors’ keywords

2. **EMBASE**

Embase <1974 to 2022 November 30>

1	("Maternal Death Surveillance and Response" or MDSR or "Maternal and perinatal Death Surveillance and Response" or MPDSR).ti,ab,kf.	87

2	((maternal death or perinatal death or neonatal death) adj2 (surveillance or response or review or reporting or audit)).ti,ab,kf.	318

3	1 or 2	332

4	limit 3 to yr="2010 -Current"	283

Link: <https://ovidsp.ovid.com/ovidweb.cgi?T=JS&NEWS=N&PAGE=main&SHAREDSEARCHID=34Jk0WzHj0jwlt7Puxf3lC20QZ01vYDRSk0k5CDlSWmp4kaAoTHASupFf1pmCdphP>

3. **Web of Science**

"Maternal Death Surveillance and Response" OR "MDSR" OR "Maternal and perinatal Death Surveillance and Response" OR "MPDSR" (Topic) OR ("maternal death") NEAR/1 ("surveillance" OR "response" OR "review" OR "reporting" OR "audit") (Topic) OR ("perinatal death") NEAR/1 ("surveillance" OR "response" OR "review" OR "reporting" OR "audit") (Topic) OR ("neonatal death") NEAR/1 ("surveillance" OR "response" OR "review" OR "reporting" OR "audit") (Topic)

Hits: 239

Comments:
Topic = search words must be in either title, abstract or authors’ keywords, or databases “Keywords PLUS”

Link: <https://www.webofscience.com/wos/woscc/summary/6b7db54b-d435-40fd-847b-80b543ed6f19-62dff96a/relevance/1>

4. **PubMed Central**

"Maternal Death Surveillance and Response" or MDSR or "Maternal and perinatal Death Surveillance and Response" or MPDSR

Hits: 314

Link: [https://www.ncbi.nlm.nih.gov/pmc?term=((%22Maternal%20Death%20Surveillance%20and%20Response%22%20or%20MDSR%20or%20%22Maternal%20and%20perinatal%20Death%20Surveillance%20and%20Response%22%20or%20MPDSR))%20AND%20(%222010%2F01%2F01%22%5BPublication%20Date%5D%20%3A%20%223000%22%5BPublication%20Date%5D)](https://www.ncbi.nlm.nih.gov/pmc?term=(("Maternal Death Surveillance and Response" or MDSR or "Maternal and perinatal Death Surveillance and Response" or MPDSR)) AND ("2010%2F01%2F01"%5BPublication Date%5D %3A "3000"%5BPublication Date%5D))

5. **JSTOR**

Maternal Death Surveillance and Response OR MDRS OR Maternal and perinatal Death Surveillance and Response OR MPDSR

Hits: 21

Link: [https://www.jstor.org/action/doAdvancedSearch?group=none&q0=%22Maternal+Death+Surveillance+and+Response%22&q1=%22MDSR%22&q2=%22Maternal+Death+Surveillance+and+Response%22&q3=%22MPSDR%22&q4=&q5=&q6=&sd=2010&ed=2023&pt=&isbn=&f0=all&c1=OR&f1=all&c2=OR&f2=all&c3=OR&f3=all&c4=AND&f4=all&c5=AND&f5=all&c6=AND&f6=all&acc=on&la=eng+OR+en](https://www.jstor.org/action/doAdvancedSearch?group=none&q0="Maternal+Death+Surveillance+and+Response"&q1="MDSR"&q2="Maternal+Death+Surveillance+and+Response"&q3="MPSDR"&q4=&q5=&q6=&sd=2010&ed=2023&pt=&isbn=&f0=all&c1=OR&f1=all&c2=OR&f2=all&c3=OR&f3=all&c4=AND&f4=all&c5=AND&f5=all&c6=AND&f6=all&acc=on&la=eng+OR+en)

6. **HeinOnline**

Search key: "Maternal Death Surveillance and Response" OR "MDRS" OR "Maternal and perinatal Death Surveillance and Response" OR "MPDSR"

Hites: 690 results.

- All are not included. As it does not result in relevant materials, even if they were listed by relevance

Link: [https://heinonline.org/HOL/LuceneSearch?terms=%28%28%22Maternal+Death+Surveillance+and+Response%22%29+OR+%22MDSR%22+OR+%22Maternal+and+perinatal+Death+Surveillance+and+Response%22+OR+%22MPDSR%22%29&collection=journals&searchtype=advanced&typea=text&tabfrom=&other_cols=yes&submit=Go&sendit=](https://heinonline.org/HOL/LuceneSearch?terms=(("Maternal+Death+Surveillance+and+Response")+OR+"MDSR"+OR+"Maternal+and+perinatal+Death+Surveillance+and+Response"+OR+"MPDSR")&collection=journals&searchtype=advanced&typea=text&tabfrom=&other_cols=yes&submit=Go&sendit=)

**7 Oria (University of Bergen’s search engine)**

Search Key: "Maternal Death Surveillance and Response" OR "MDRS" OR "Maternal and perinatal Death Surveillance and Response" OR "MPDSR"

Hits: 288.

- Only the first 100 were taken, as the list after that was irrelevant
- Time frame for this was ‘last 10 years’ as it does not allow further specification

Link: [https://bibsys-almaprimo.hosted.exlibrisgroup.com/primo-explore/search?query=any,contains,%22Maternal%20Death%20Surveillance%20and%20Response%22,OR&query=any,contains,%22MDSR%22,OR&query=any,contains,%22Maternal%20and%20Perinatal%20Death%20Surveillance%20and%20Response%22,OR&query=any,contains,%22MPDSR%22,AND&pfilter=lang,exact,eng,AND&pfilter=creationdate,exact,10-YEAR,AND&tab=default_tab&search_scope=default_scope&vid=UBB&lang=en_US&mode=advanced&offset=50](https://bibsys-almaprimo.hosted.exlibrisgroup.com/primo-explore/search?query=any,contains,"Maternal Death Surveillance and Response",OR&query=any,contains,"MDSR",OR&query=any,contains,"Maternal and Perinatal Death Surveillance and Response",OR&query=any,contains,"MPDSR",AND&pfilter=lang,exact,eng,AND&pfilter=creationdate,exact,10-YEAR,AND&tab=default_tab&search_scope=default_scope&vid=UBB&lang=en_US&mode=advanced&offset=50)
