# Supplementary material for: Legal issues in the implementation of Maternal Death Surveillance and Response: a scoping review
Source: Health Policy Plan. 2024 Aug 3;39(9):985–99. doi: 10.1093/heapol/czae071 (PMC11474607; doi:10.1093/heapol/czae071)
Supplement: czae071_Supp [file czae071_supp.zip › Supplementary File 3_Record Index.doc]

**Supplementary File 3:** Record index: list and numbers

#1. Abebe B *et al.* 2017. 'We identify, discuss, act and promise to prevent similar deaths': A qualitative study of Ethiopia's Maternal Death Surveillance and Response system. *BMJ Global Health* **2**(2): e000199.
#2. Abouchadi S *et al.* 2022. Eight-year experience of Maternal Death Surveillance in Morocco: Qualitative study of stakeholders' views at a subnational level. *BMC Public Health* **22**(1), 2111.
#3. Agaro C *et al.* 2016. The conduct of maternal and perinatal death reviews in Oyam District, Uganda: A descriptive cross-sectional study. *BMC Women's Health* **16**: 38.
#4. Armstrong CE *et al.* 2014. Strengths and weaknesses in the implementation of Maternal and Perinatal Death Reviews in Tanzania: Perceptions, processes and practice. *Tropical Medicine & International Health* **19**(9): 1087-95.
#5. Bongajum AY *et al.* 2021. Strengthening Maternal Death Surveillance Systems for evidence-based decision making in Sub-Saharan Africa: The case of the Center Region in Cameroon. *International Journal of MCH & AIDS* **10**(2): 258-68.
#6. Boyi HC *et al.* 2022. “So hard not to feel blamed!”: Assessment of implementation of Benin’s Maternal and Perinatal Death Surveillance and Response Strategy from 2016–2018. *Int J Gynaecol Obstet* **158**: 6-14.
#7. Cahyanti RD, Widyawati W, Hakimi M. 2021. "Sharp downward, blunt upward": district maternal death audits' challenges to formulate evidence-based recommendations in Indonesia - a qualitative study. *BMC Pregnancy & Childbirth* **21**(1): 730.
#8. Compaore R *et al.* 2022. Transitioning to the Maternal Death Surveillance and Response system from Maternal Death Review in Ghana: Challenges and lessons learned. *International Journal of Gynaecology & Obstetrics* **158** **Suppl 2**: 37-45.
#9. Goodman D *et al.* 2013. Revival of a core public health function: State- and urban-based maternal death review processes. *Journal of Women's Health* **22**(5): 395-8.
#10. Kinney MV *et al.* 2021. Maternal and Perinatal Death Surveillance and Response in Low- and Middle-Income Countries: A scoping review of implementation factors. *Health Policy & Planning* **36**(6): 955-73.
#11. Kinney MV. *et al.* 2020. "It might be a statistic to me, but every death matters.": An Assessment of facility-level Maternal and Perinatal Death Surveillance and Response Systems in Four Sub-Saharan African Countries. *PLoS ONE* **15**(12): e0243722.
#12. Kodan LR *et al.* 2021. From passive surveillance to response: Suriname's efforts to implement Maternal Death Surveillance and Response. *Global Health Science & Practice* **9**(2): 379-89.
#13. Kouanda S *et al.* 2022. Analysis of the implementation of maternal death surveillance and response in Chad. *International Journal of Gynaecology & Obstetrics* **158 Suppl 2**: 67-73.
#14. Lardi M *et al.* 2019. Implementing Maternal Death Surveillance and Response System: Lessons learned from the Arab States Region. *Acta Scientific MEDICAL SCIENCES* **5**(12): 166-78.
#15. Melberg A *et al.* 2019. 'Maternal deaths should simply be 0': Politicization of maternal death reporting and review processes in Ethiopia. *Health Policy & Planning* **34**(7): 492-8.
#16. Melberg A *et al.* 2020. Juridification of maternal deaths in Ethiopia: A study of the Maternal and Perinatal Death Surveillance and Response (MPDSR) System. *Health Policy & Planning* **35**(8): 900-05.
#17. Mukinda FK *et al.* 2021. Practice of Death Surveillance and Response for Maternal, Newborn and Child Health: A framework and application to a South African health district. *BMJ Open* **11**(5): 1-13.
#18. Mutsigiri-Murewanhema F *et al.* 2017. Evaluation of the Maternal Mortality Surveillance System in Mutare District, Zimbabwe, 2014-2015: A cross sectional study. *The Pan African Medical Journal* **27**: 204.
#19. Naik SS *et al.* 2020. Quality assessment of Maternal Death Review: A pilot study in 10 high priority districts of Odisha State, India. *Indian Journal of Community Medicine* **45**(2): 184-8.
#20. Russell N *et al.* 2022. Implementation of Maternal and Perinatal Death Surveillance and Response (MPDSR) in humanitarian settings: Insights and experiences of humanitarian health practitioners and global technical expert meeting aattendees. *Conflict & Health* **16**(1): 23.
#21. Said A *et al.* 2021. Mismatched ambition, execution and outcomes: Implementing Maternal Death Surveillance and Response system in Mtwara Region, Tanzania. *BMJ Global Health* **6**(5): 5.
#22. Scott H, Dairo A. 2015. Maternal Death Surveillance and Response in East and Southern Africa. *Journal of Obstetrics & Gynaecology Canada: JOGC*, **37**(10): 915-21.
#23. Shellhaas C, Conrey E. 2018. State-based review of maternal deaths: The Ohio experience. *Clinical Obstetrics & Gynecology* **61**(2): 332-9.
#24. Shittu O, Kinney MV. 2017. *Assessment of Maternal and Perinatal Death Surveillance and Response implementation in Nigeria*. [https://www.healthynewbornnetwork.org/hnn-content/uploads/Nigeria-national-mpdsr-assessment-report-final.pdf (25](https://www.healthynewbornnetwork.org/hnn-content/uploads/Nigeria-national-MPDSR-Assessment-report-FINAL.pdf (25) November 2023)
#25. Singh S *et al.* 2015. Community-based Maternal Death Review: Lessons learned from ten districts in Andhra Pradesh, India. *Maternal & Child Health Journal* **19**(7): 1447-54.
#26. Smith H *et al.* 2017a. Implementing Maternal Death Surveillance and Response: A review of lessons from country case studies. *BMC Pregnancy & Childbirth* **17**(1): 233.
#27. Smith H. *et al.* 2017b. Implementing Maternal Death Surveillance and Response in Kenya: Incremental progress and lessons learned. *Global Health Science & Practice* **5**(3): 345-54.
#28. WHO. 2014. *Maternal Death Review in Selected Countries of South-East Asia Region*: World Health Organization.
#29. WHO. 2016a. *Time to Respond: A Report on the Global Implementation of Maternal Death Surveillance and Response*. World Health Organization.
#30. Willcox ML *et al.* 2023. Maternal and Perinatal Death Surveillance and Response: A systematic review of qualitative studies. *Bull World Health Organ* *101*(1): 62-75g.
#31. Yameogo WM E *et al.* 2022. Assessment of the Maternal Death Surveillance and Response implementation process in Burkina Faso. *International Journal of Gynaecology & Obstetrics* **158** **Suppl 2**:15-20.
