# Supplementary material for: Legal issues in the implementation of Maternal Death Surveillance and Response: a scoping review
Source: Health Policy Plan. 2024 Aug 3;39(9):985–99. doi: 10.1093/heapol/czae071 (PMC11474607; doi:10.1093/heapol/czae071)
Supplement: czae071_Supp [file czae071_supp.zip › Supplementary File 4_Key Characterstics.doc]

**Supplementary File** **4:** Key characteristics of selected records

| Assigned  Record # | Author(s) and year | Country (ies) of study/focus | Scale of study | Focus of MDSR cycle | Research design |
| --- | --- | --- | --- | --- | --- |
| #1. | Abebe *et al*. 2017 | Ethiopia | National | All | Qualitative |
| #2. | Abouchadi *et al*. 2022 | Morocco | Subnational | All | Qualitative |
| #3. | Agaro *et al*. 2016 | Uganda | Subnational | All | Mixed |
| #4. | Armstrong *et al*. 2014 | Tanzania | National | All | Qualitative |
| #5. | Bongajum *et al*. 2021 | Cameroon | Subnational | All | Qualitative |
| #6. | Boyi Hounsou *et al*. 2022 | Benin | National | All | Mixed-method |
| #7. | Cahyanti *et al*. 2021 | Indonesia | Subnational | Mainly  response | Qaulitative |
| #8. | Compaore *et al*. 2022 | Ghana | National and sub national | All | Mixed-method |
| #9. | Goodman *et al*. 2013 | USA | Subnational | Mainly review | Qaulitative |
| #10. | Kinney *et al*. 2020 | Multi-country (Sub-Sahara) | National and sub national | All | Mixed-method |
| #11. | Kinney *et al*. 2021 | Multi-country  (LMIC) | National and sub national | All | Qaulitative |
| #12. | Kodan *et al*. 2021 | Suriname | National | All | Qaulitative |
| #13. | Kouanda *et al*. 2022 | Chad | National | All | Qaulitative |
| #14. | Lardi *et al*. 2019 | Multi-country (Arab region) | National | All | Qaulitative |
| #15. | Melberg *et al*. 2019 | Ethiopia | Combination of levels | All | Qualitative |
| #16. | Melberg *et al*. 2020 | Ethiopia | Combination of levels | Mainly reporting | Qualitative |
| #17. | Mukinda *et al*. 2021 | South Africa | Subnational | All | Qualitative |
| #18. | Mutsigiri-Murewanhema *et al*. 2017 | Zimbabewe | Subnational | Mainly reporting | Mixed-method |
| #19. | Naik *et al*. 2020 | India | Subnational | All | Qualitative |
| #20. | Russell *et al*. 2022 | Multi-country | National | All | Qualitative |
| #21. | Said *et al*. 2021 | Tanzania | Subnational | All | Qualitative |
| #22. | Scott & Dairo 2015 | Multi-country (Africa) | National | All | Qualitative |
| #23. | Shellhaas & Conrey 2018 | USA | Subnational | All | Qualitative |
| #24. | Shittu & Kinney 2017 | Nigeria | National | All | Qualitative |
| #25. | Singh *et al*. 2015 | India | Subnational | All | Mixed-method |
| #26. | Smith *et al*. 2017a | Kenya | National | All | Qaulitative |
| #27. | Smith *et al*. 2017b | Multi-country | National | All | Qaulitative |
| #28. | Willcox *et al*. 2023 | Multi-country | National | All | Qualitative |
| #29. | WHO 2014 | Multi-country | National | All | Qaulitative |
| #30. | WHO 2016 | Multi-country | Mainly national | All | Mixed method |
| #31. | Yameogo *et al*. 2022 | Burkina Faso | National | All | Qualitative |
