# Supplementary material for: Legal issues in the implementation of Maternal Death Surveillance and Response: a scoping review
Source: Health Policy Plan. 2024 Aug 3;39(9):985–99. doi: 10.1093/heapol/czae071 (PMC11474607; doi:10.1093/heapol/czae071)
Supplement: czae071_Supp [file czae071_supp.zip › Supplementary File 5_Review result details.doc]

**Supplementary File 5:** Review result details

| Topic/domain | # | Main Findings |
| --- | --- | --- |
| Data | #2 | - Lack of confidentiality affects engagement with stakeholders, sharing results, and implementing recommendations. |
|  | #4 | - Guidelines provide for principles of confidentiality and anonymity, but the names of the deceased and the facility were on the reporting form. - Lack of anonymity (of the deceased and health facility) in the report to national level contributed to poorer dialogue during case reviews. |
|  | #5 | - WhatsApp is used to facilitate communication. |
|  | #7 | - Reviewers reveal the identity of health workers and institutions to the public; reviewers acting as judges. |
|  | #8 | - Use of SMS, telephone, and paper reports for notification. - Lack of secure storage for confidential maternal death data. - No formal mechanism for sharing maternal health data between the surveillance system and CNMH service. |
|  | #9 | - Problem of accessing medical records among the challenges, as 90% of the systems reviewed use data linkage. |
|  | #10 | - A paper-based system was used in all countries surveyed. |
|  | #12 | - There is an understanding that audits are undertaken with ‘no name, no shame principle.’ Yet, it is not complied with. - Laws are suggested to strengthen implementation. |
|  | #14 | - IT software is used in all the countries covered to manage MDSR-related data. |
|  | #20 | - Paper-based systems allow deaths to be more easily concealed. |
|  | #21 | - Notification is carried out through WhatsApp groups and SMS, even to a regional level. |
|  | #22 | - Concern for the anonymization of the data begins after a local review is undertaken (standard operation of the MDSR) - After data is collected & is entered into health information database, ‘all records are destroyed.’ |
|  | #23 | - Data is identified through pregnancy checkbox, through requests (based on specific authority to do so), and the signing of data-sharing agreements with various programmes (linkage). |
|  | #26 | - Photocopies of original case notes are made and sent to the secretariat office, with the original notes remaining on site. - Case notes are centrally collated and anonymized, and subsequently sent to assessors for review. |
|  | 27 | - All information received by the committee is completely anonymized and destroyed once reports are published. |
|  | #29^[1]^ | - The identity of the deceased cannot be completely anonymous in death reviews. |
| People | #1 | - Failure to report to avoid reviewers, ‘since a review is often considered as a type of investigation to blame.’ - Widespread fear that an increase in reported maternal deaths could lead to legal or disciplinary actions against health workers. - Focus on institutional delivery induces fear of reporting community maternal deaths (by community members). - Health professionals press for legal protection. - Strong political will to reduce maternal mortality fosters the fear of blame and legal repercussions. |
|  | #2 | - The blame culture is a major source of concern impeding implementation. - Punitive malpractice associated with the review system is reported. - Incidents where health managers refer to health workers as murderers during reviews. - Midwives are unable to speak openly about the causes and circumstances of maternal deaths. - Fear of maternal complications or blame for a maternal death led to unnecessary referrals to the next level of care. - Review members blamed deaths on factors that were frequently beyond their control, leading to controversies with managers. |
|  | #3 | - Fear of litigation by the government listed among the challenges health workforce faces. - Community members tend to blame death on facilities and deny that they sought help late. |
|  | #4 | - Informants reported instances of blame; people fear being blamed, fear that they will be considered unskilled (midwife). |
|  | #6 | - Health workers’ fear of blame is among the principal causes of under-notification. - Fear of blame is among the reasons for a low death review completion rate. |
|  | #7 | - Tendency to blame lower health workers for mismanagement ‘sharp downwards, blunt upwards.’ |
|  | #8 | - Providers fear of blame identified as a challenge, and training as a solution. |
|  | #10 | - Measures include an oral and written warnings, reprimands, suspension and ‘other punishments.’ |
|  | #11 | - Blame culture and punitive measures against frontline health providers are recognized as barriers. - Instances include feeling threatened during review, fearing legal action or punitive repercussions, and a general culture of blame. - Possible reason: lack of clarity around the process when first implemented. - Also, findings that fear of litigation has a positive effect on the implementation. |
|  | #12 | - Anonymity and the “no blame, no shame” culture were not assured in the facilities. - Assurance against litigation is provided, but not complied with. - Legislation framework is recommended to ensure timely notification and completion of death certificates, conduct autopsy for deaths of unknown cause, as well as ensure that no litigation/disciplinary measures is initiated in relation to death audits. |
|  | #13 | - Fear of having to explain high death numbers to the Minister derive underreporting and underestimation. - Under-reporting and concealment of information for fear of repercussion is reported. |
|  | #14 | - Reasons for weak dissemination include fear of manipulation by media and medical associations. |
|  | #15 | - Fear of blame, including for failing to prevent the death among frontline workers, is reported. - Fear of external investigation and exposure of medical malpractice hinder reporting. - Litigation, as a major concern for health workers, affected how causes of death is reported. - Among the ways of deflecting responsibility: - Selective reporting, - Considering such deaths as due to late arrival or infrastructural factors as unpreventable, - Tendency to blame the women themselves, and - Rushed and unassisted referral (defensive). |
|  | #16 | - The number of malpractice accusations against obstetricians is on the rise. - Instances of police questioning or detaining obstetricians for malpractice allegations recorded. - Courts considered as being focused on individuals, not hospitals, reflecting lack of health workers protection. - Blame is directed towards individual health workers, not institutions. - While doctors were concerned with formal legal accusations, lower-level health workers were worried about informal sanctions from local politicians and the health administration. - Increased awareness about maternal death precipitating the increase and fear of litigation. - Some defense mechanisms used: - Health workers’ tendency to classify maternal deaths as non-preventable, - Defensive referral, - Instances where health workers chose not to intervene (stop bleeding) in fear of medico-legal consequences, - The introduction of malpractice indemnity insurance. |
|  | #17 | - ‘No name, no blame’ approach is followed in most instances as stipulated in guiding documents, increasing interpersonal trust. - In certain instances, a culture of organizational blame and punishment is noted. - Certain types of reviews resulted in intensive scrutiny, seeking to assign individual responsibility and frequently triggering sanction and punitive action. - Suspensions, referral to labour offices, litigation, and court cases involving front-line professionals is reported. |
|  | #18 | - Fear of blame is among the reasons for not reporting maternal death. - To address this, explaining the repercussions of the problem and advice/support in orientation sessions is recommended. |
|  | #19 | - Fear of punitive action among health staff as a weakness of the system. - Blaming junior doctors for inaccurate reports observed. - Non-confidentiality is identified as a threat. - Under-reporting is observed in the great majority of districts studied. - Particular problem relates to early pregnancies and deaths related to illegal abortion. |
|  | #20 | - In an environment where confidentiality and anonymity are challenging, health workers are blamed for death, risking their security. - Underreporting is driven by concerns about ‘negative consequences’ or facility reputational damage. - Punitive approach to Traditional Birth Attendants discourages reporting of community deaths. - Refusal of admission in referral facilities for women appearing likely to die, apparently with the motivation of avoiding damaging mortality statistics is reported. - Underreporting and misclassification of abortion-related deaths in legally restrictive settings. - The fear leading to reluctance to openly discuss causes of death and provide useful recommendations. |
|  | #21 | - Fear of blame during meetings caused health providers to avoid attendance. - Instances of reprimanding employees based on findings from the meetings. - This resulted in the falsification of documents, late notification, and concealing cases of death. - Managers breached the confidentiality of the meetings to counter weak attendance during reviews. - Breach of confidentiality and ‘the blame culture’ seriously affected the review process. - To counter the blame culture, holding managers to be ‘true to their promise of upholding confidentiality’ is recommended. |
|  | #22 | - Blame being placed on the ‘lower levels’, including the community. |
|  | #23 | - Maternal death review was practiced in the 1970s and 1980s but was ended due to «concerns about malpractice litigation and liability.' - To facilitate death notification and mitigate delays caused civil or criminal litigations associated with access to records, checkbox was introduced. - Review meetings begin with an overview of the process, a reminder of definitions and confidentiality. |
|  | #24 | - Some facilities instituted disciplinary action against staff who were observed to have erred during service delivery to deceased patients. - Blame was reported to be more severe when staff from multiple disciplines took part. - Some staff members are reprimanded and receive other punishments. - When negligence is ascribed to a health worker, the culprit is referred to a disciplinary committee. - ‘Appropriate laws that will shield MPDSR implementation from abuse and litigation’ is called for. - The Federal government is recommended to issue directives to Tertiary Health Centers (THCs) to commence MPDSR. - A facility-based code of conduct is recommended to codify the ethos of the non-punitive nature of the process that the national guideline stipulates. - Introducing legislation to entrench the “no name, no blame” atmosphere for MPDSR was recommended. |
|  | #25 | - Fear of blame among health staff was identified as a weakness, while the lack of confidentiality in the process was seen as a threat. - More underreporting was observed in areas with higher MMR. - Staff attribute under/nil reporting to fear of punitive action – suggested correction orientation and sensitization. - Observation that maternal deaths due to illegal abortion were missing. - The review committee tends to blame the private sector and subordinate staff for failure. - Lack of participation by the private sector is identified as a challenge. - Private institutions tend to refer very sick women to district-level government institutions in the last stages.’ - As a policy, review details were not shared with media. |
|  | #26 | - No name, no shame approach was included in response to underreporting, two years after notification was made mandatory, only half of the estimation was being reported. - Assuring health providers that the information obtained as part of MPDSR will not be used for litigation is recommended. |
|  | #27 | - There is recognition that without an adequate legal framework and sensitization of health workers to the ‘blame free’ principle, the government plans to progress the maternal death review system may stall. |
|  | #28 | - While many demanded for legal protection against the use of MDSR data in litigation, only South Africa had enacted such a law, ratified by judicial bodies. - Some reported harmful outcomes of a vicious cycle, characterized by lack of legal protection, include: - staff shortage, as more became afraid to work in the labour ward, - some taking several weeks off after an upsetting review, - junior doctors deterred from choosing obstetrics as a career, - Some practices of defensive medicine, such as inappropriate referral of unstable patients at high risk, - Refusal to admit referred women who seemed likely to die, - Fear of blame and negative consequences were the most pervasive mechanisms for a vicious cycle. - Results include disengagement, underreporting, inaccurate data, inadequate formulation of solutions, and avoidance of responsibility. - Main drivers: insufficient confidentiality or anonymity. - Deliberate falsification and misclassification of death to avoid blame or reputational damage is reported. - Blame culture, in the form of health workers and families being questioned about a death, is exacerbated in countries under an authoritarian system, where confidentiality is not guaranteed, and where families had no other avenues than litigation. - Completely removing blame is almost impossible, as negligence is going to be uncovered and will need to be tackled. - The separation may be impossible to achieve in practice, where many facility heads are also heads of review committees/maternal units. |
|  | #29 | - Maternal deaths due to abortion are not reported and not captured for a review. - Underreporting due to midwives deliberately not reporting for fear of being blamed for death during audit. - Underreporting is particularly severe in cases of women dying in another administrative area. - Birth attendants tend to refuse to answer questions, as they fear the health department might sue them. - Particular difficulties in obtaining information from the private sector, especially unlicensed facilities. - Fear of punitive action among health-care providers leads to under-reporting or demotivation. - ‘Fear of punitive action and repercussion from the community, there is reluctance from health functionaries to conduct reviews. - Fear of being sued or other punitive action being real, ‘proper briefing, training and motivating all staff involved in the audit’ is suggested as a solution. - Fear of possible litigation is significant in three of the study countries. - Lack of legal immunity is cited as a weakness. - Reported consequences include the exclusion of private sector experts and lay people in review committees. |
|  | #30 | - Legal provisions related to confidentiality and medical liability will help to allay fears about participating in maternal death review. - Evidence for a blame culture emerged in several instances. - Result: Sometimes led to falsification of medical documents to conceal the true circumstances of death. - Protect health professionals from recrimination and reprisals by assuring legal protection and the principles of anonymity and confidentiality. |
|  | #31 | - Some providers perceived maternal death as a failure of the facility or that the health center was solely responsible. - Intentional misreporting of maternal death in case of illegal abortion. - Recommendations include providing ‘better information for health providers on the ‘no blame’ principle in MDSR. |
| Use of findings | #1 | - Perceived fear that maternal death can be related to professional malpractice, negligence, or failure to comply with policy. |
|  | #2 | - Local authorities use reports, mainly death preventability rates, to attribute fault to the health care system and professionals. |
|  | #7 | - Perception that equates the review process with a ‘disciplinary process.’ |
|  | #10 | - Connection between professional disciplinary action and MPDSR activities reported in 23 facilities. |
|  | #14 | - Need for legal guarantees to prevent the results of the review from being used for trial purposes is recommended. |
|  | #15 | - The number on maternal deaths being part of evaluation for managers reinforces interference with reporting. |
|  | #16 | - A sense that the MPDSR system, contrary to its objective, is misused to identify individuals. |
|  | #18 | - Some health workers see the process as a fault-finding exercise. |
|  | #19 | - Findings of the review process were kept strictly confidential, and not shared with any media. |
|  | #20 | - Reviews evolving into human resource and are linked to disciplinary processes. - In settings with less trained staff, MPDSR can be easily misunderstood as a tool for disciplining staff. - Boundaries between the review system and human resource management blurred, including linking information. |
|  | #21 | - MDSR information is used to punish health providers. |
|  | #22 | - Misconception about confidentiality and accountability as major challenges and weaknesses. - Misconception regarding discipline and an understanding that the maternal death review was frequently punitive. |
|  | #24 | - The “need to have legislation in place to entrench ‘no name, no blame’ atmosphere for MPDSR and insulate it from litigation processes” is recommended. |
|  | # 26 | - Facilities showed reluctance to share the note on account of … ‘lack of trust and suspicion about what the case notes will be used for, and, linked to this, the fear of blame and follow-up action against individuals because of the information contained within the reports…’ |
|  | #27 | - SA- relevant judicial bodies have ratified the process so that data collected, and review forms used in the process cannot be used in litigation or disciplinary procedures. |
|  | #28 | - Learning environments assure confidentiality, anonymity, and separation of blame or a disciplinary process. - One reason for the blame culture, the failure to separate the death review from disciplinary procedures. |
|  | #31 | - Some avoid notifying maternal death to avoid receiving a review team, since ‘it is often considered as a type of investigation to blame.’ |
| Legal regulation | #1 | - Maternal death became a notifiable event in 2014. - Integrating the MDSR into the health information system (PHEM) and assigning officers to coordinate the MDSR activities with no clarity created confusion and neglect during implementation. |
|  | #2 | - Recommended that ethical and legal issues be anticipated and addressed beforehand. - Achieving the right balance between “no blame” and accountability is challenging.' and a call for 'just culture.' |
|  | #3 | - Maternal death became a notifiable event in 2008. - Despite its introduction through a presidential directive, implementation remained irregular. |
|  | #4 | - No clear lines of accountability were made explicit, complicating the response cycle. - Ambiguity in the purpose of MDSR is a challenge. |
|  | #5 | - Notification of deaths and the creation of district review committees was mandated by a ministerial text. - Lack of regulations enforcement strategies for the application of national guidelines was a major challenge. - Absence of burial permits and poor vital registration contribute to poor capturing of community deaths. |
|  | 6 | - Notification is mandatory. - MPDSR committee in each health district is established by decree. |
|  | #7 | - A decree established MDA committees. - MoU signed between district health facilities and government, requiring health facilities to notify maternal death to the committee – often unenforced. - Confusion in authority hampers enforcement of recommendations. |
|  | #8 | - Certain hospitals were not aware of their obligations to notify maternal death. |
|  | #9 | - The existence of legislation is used as a selection criterion for a functioning maternal death review system. - Only 25% of assessed maternal death review processes have authorizing legislative support. - Only in half of them were committee members offered immunity from legal actions. |
|  | #11 | - The absence of a strong MPDSR legal framework across all levels is a factor driving fear. - Benefits of having national guidelines include informing the implementation process. |
|  | #13 | - Obligation to notify maternal death and institutionalization of the review system is regulated by ministerial order. - Political commitment (involvement of the head of state and enactment of laws) did not guarantee the continuity of the MDSR – with lack of engagement by national health providers and other decision-makers as a major reason. - Three circulars on the implementation of the MDSR are identified. |
|  | #14 | - Maternal death is made a notifiable event through ministerial decrees and circulars. - Legislation does not include the private sector. |
|  | #15 | - Maternal death forms part of public health emergencies, thus notifiable. |
|  | #16 | - Maternal death is classified as a public health emergency. |
|  | #17 | - There is mandatory registration of maternal death. |
|  | #21 | - MDSR-related decisions, including committee membership, are decided by the facility Director. - The MDSR is detached from the notification system and quality improvement teams in the facilities concerned. - Lack of direct engagement with community members is listed as a challenge. |
|  | #22 | - Lack of legal framework for MDSR as a major challenge and weakness. - In some countries, there is a reported lack of a legal framework for the notification of maternal deaths. - Underreporting is reported in countries where maternal death is a notifiable event. - Private health facilities do not always disclose the cause of death. - In some instances, no linkage between MDSR and integrated disease surveillance and response exist. - In South Africa, maternal death is notifiable by law. - Little attention is given to determinants of health and how they affect maternal wellbeing. |
|  | #23 | - Ohio Pregnancy-Associated Mortality Review Board includes 'Legal services including risk management.' - Law that supports the request states “reports of births and deaths shall be subjects of study by the director of health of the state of Ohio.” - The state does not currently have legislation specific to maternal death reviews. - Mandatory maternal death reporting was introduced in 2012 as a result of the exercise. |
|  | #24 | - MDSR, starting with maternal death review, was introduced in 2013 by the FMoH, driven by need and WHO TG (2013). |
|  | #25 | - In 2011, the government rolled out guidelines for mandatory implementation of maternal death reviews in both facilities and communities. - Data is compiled and investigated by district maternal death review, chaired by district administration head. |
|  | #26 | - The government of Kenya made maternal death notification mandatory in response to high maternal deaths. - a national committee for MPDSR "was inaugurated by the Cabinet Secretary for Health after 18 months of stakeholder consultations. - Some pointed out that there is no legal mandate to retrieve case notes, so the process is entirely based on goodwill. - A bill that provides a legal framework for MPDSR was hoped to be passed. - This is needed to enforce maternal death notification by law. |
|  | #27 | - The law on notification of maternal deaths in 1997 was a precursor to the national review system introduced. - Transition to MPDSR was initiated with awareness creation activities held at statewide or facility meetings. - The hospital’s chief medical director, director of clinical services, or the most senior medical officer usually chaired the MPDSR committees. |
|  | #28 | - In the absence of legal protection, the next best context was an audit charter (signed by review members). - Telling participants that the process was blame-free was insufficient to allay fears when senior managers were present or litigation against health workers was increasing. - The absence of mandatory reporting contributes to under-reporting of deaths. |
|  | #29 | - Notification and conducting a postmortem on all maternal deaths is a legal requirement (Sri Lanka). |
|  | #30 | - Incomplete/inadequate legal frameworks, among the case study insights. - Making maternal death a notifiable event in all countries is recommended. |

1. This report consists of five separate parts, covering studies carried out in five countries (India, Indonesia, Myanmar, Nepal, and Sri Lanka) and a chapter that presents a synthesis. We used the synthesis chapter for this review.
